# Diabetes burden attributable to air pollution from 1990~2021 and the future trends: a population-based study

**DOI:** 10.3389/fendo.2025.1475822

**Published:** 2025-04-08

**Authors:** Qingsong Mao, Xiaoyi Zhu, Xinyi Zhang, Yuzhe Kong

**Affiliations:** ^1^ Hepatobiliary Pancreatic Surgery, Banan Hospital Affiliated of Chongqing Medical University, Chongqing, China; ^2^ Xiangya School of Medicine, Central South University, Changsha, China; ^3^ College of Education, Wenzhou University, Wenzhou, China

**Keywords:** diabetes, air pollution, mortality forecasting, epidemiology, disease burden

## Abstract

**Background:**

This investigation explores the worldwide impact of diabetes burden associated with air pollution, drawing on data from the Global Burden of Disease Study 2021.

**Method:**

The influence of air pollution on diabetes burden was analyzed at global, regional, and national levels. The study considered variations across age groups and genders and explored the relationship between disease impact and the Socio-Demographic Index (SDI). Additionally, an ARIMA model was employed to predict the future incidence of diabetes burden related to air pollution until 2050.

**Result:**

In 2021, approximately 281.91 thousand fatalities and 12.90 million disability-adjusted life years were attributed to diabetes burden due to air pollution, featuring an age-standardized mortality rate (ASMR) of 3.3234 (95% UI, 1.9549–4.6634) and an age-standardized DALY rate (ASDR) of 148.9167 (95% UI, 86.5013–224.9116) per 100,000 individuals. There was a noticeable escalation in the disease burden over the period studied. The most severe effects were noted in individuals aged 60 and above. The data also revealed a higher disease burden among males. Forecasting suggests that while low SDI regions might see a decrease in death rates, lower-middle SDI areas could face an increase in standardized mortality rates. On a national scale, Russia, Mexico, and several African nations are predicted to experience rising diabetes burden attributable to air pollution mortality rates and age-standardized mortality rates from now to 2050. South Asia and Africa are anticipated to witness substantial growth in age-standardized death rates compared to other areas.

**Conclusion:**

The results provide essential insights for developing preventive strategies for diabetes burden and measures to mitigate air pollution.

## Graphical Abstract

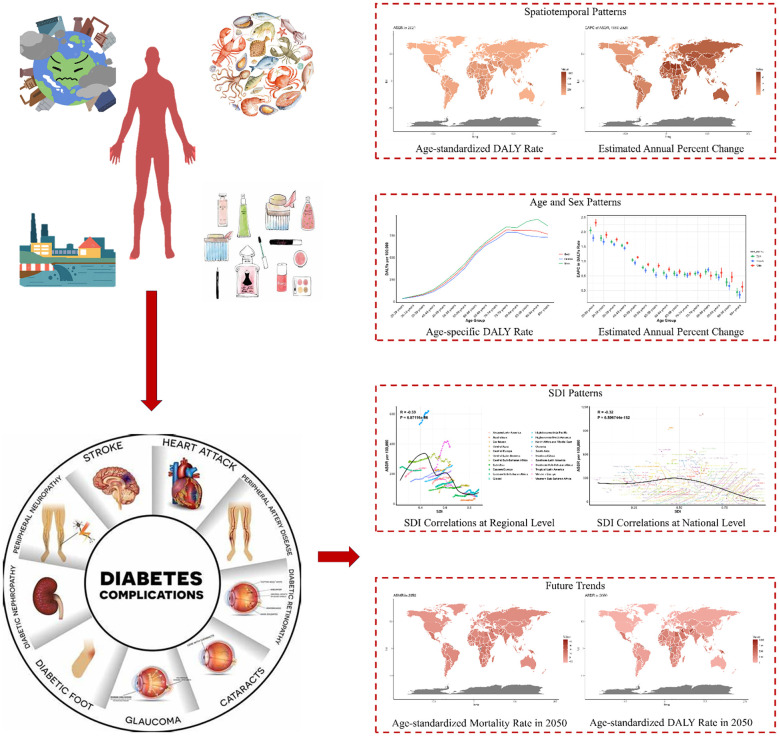

## 1 Introduction

Diabetes, a persistent medical condition, interferes with how the body processes food into energy. It often emerges in individuals with obesity, which impairs glucose management and heightens insulin resistance, elevating diabetes risk (1). Approximately 90% of type 2 diabetes cases are linked to excess weight or obesity, and the prevalence of obesity-induced impaired glucose tolerance is projected to climb to 420 million globally by 2025 (2). The simultaneous presence of diabetes and obesity, known as diabesity, can intensify cardiometabolic disorders and is emerging as a significant global health challenge amid the obesity crisis (3). Understanding the modifiable risk factors is essential for the large-scale control and prevention of diabesity.

Environmental hazards, particularly fine particulate matter (PM2.5), pose significant global health risks. Research indicates that PM2.5 exposure correlates with higher risks of both obesity (4, 5) and diabetes (6, 7). Yet, the impact of PM2.5 on diabesity remains unexplored. Animal studies suggest that PM2.5 exposure can trigger insulin resistance and systemic inflammation (8, 9), leading to obesity (10) and disrupted glucose tolerance, changes in adipokine levels, and mitochondrial dysfunction, eventually causing diabetes (11). Recent findings from the UK Biobank also demonstrate a more pronounced link between PM2.5 exposure and diabetes among obese individuals. Despite this, global studies specifically addressing the impact of air pollution on diabetes mellitus are lacking.

While well-established risk factors like obesity, lack of physical activity, and hereditary traits are recognized (12, 13), emerging evidence suggests a critical impact of environmental contributors, particularly air pollution, on the development of type 2 diabetes mellitus (T2DM). A comprehensive review and meta-analysis involving over 1.7 million individuals from 16 different studies has identified a robust link between sustained exposure to fine particulate matter (PM2.5) and a heightened risk of T2DM. Notably, an increase of 10 µg/m3 in PM2.5 levels corresponds to a 9% rise in the incidence of T2DM (14, 15). This highlights the urgency of exploring the underlying mechanisms connecting air pollution and T2DM, and the imperative to devise effective preventive measures.

However, previous studies didn’t analyze the overall disease burden of diabetes burden attributable to air pollution. Thus, this study assesses how air pollution affects the global burden of diabetes burden by analyzing mortality and disability-adjusted life years (DALYs) trends across various demographics and socio-economic indicators from 1990 to 2021. Future trends are also projected using the autoregressive integrated moving average (ARIMA) model, supported by earlier studies (12, 13). These insights aim to support the prevention of diabetes burden and the management of environmental pollution.

## 2 Method

### 2.1 Study population

Our research utilized data from the Global Burden of Disease Study (GBD) 2021, available at http://ghdx.healthdata.org/gbd-results-tool. The GBD 2021 encompasses a comprehensive assessment of 369 diseases and injuries, along with 87 risk factors, spanning 204 countries from 1990 to 2021 (14).

The methodologies adopted to evaluate the diabetes burden are described in external references (15, 16). This summary outlines the methods employed in the GBD 2021. Data on diabetes mellitus mortality were sourced from vital registration systems, surveillance systems, and verbal autopsy reports. Adjustments were made to this vital registration data to enhance precision and to correct data deficiencies and misclassification issues (17). These corrections were incorporated into the Cause of Death Ensemble model (CODEm), which generated localized, annual estimates of diabetes-related deaths by age and gender (16, 18). A comparative risk assessment was also conducted to identify principal risk factors for diabetes mellitus. The population attributable fraction (PAF) was computed to assess the extent of air pollution’s contribution to the diabetes burden. The estimates of diabetes mellitus mortality and disability-adjusted life years (DALYs) related to air pollution were derived by applying these specific PAFs to the mortality and DALY statistics by location, year, age, and gender (14). DALYs represent an aggregate measure of disease burden, integrating years of life lost due to premature death (YLLs) and years lived with disability (YLDs). YLLs were calculated by multiplying the number of deaths due to lower respiratory infections (LRIs) within each age group by the group’s residual life expectancy. YLDs were calculated by multiplying the prevalence of LRIs by the disability weights adjusted for severity (17). The Socio-Demographic Index (SDI), ranging from 0 (lowest) to 100 (highest), was determined based on factors such as the total fertility rate for those under 25 years old (TFU25), average educational level for those over 15 (EDU15+), and adjusted per capita income. Based on the SDI, the 204 nations and territories were classified into five categories: low SDI, low-middle SDI, middle SDI, high-middle SDI, and high SDI (17).

### 2.2 Statical analysis

Age-standardized rates (ASR) were employed to adjust mortality and DALY figures for countries with diverse age structures and demographic profiles. We applied a linear regression to the natural logarithm of these rates, expressed as y = α+βx+ϵ, where x denotes the year, and y is the natural log of the rate. The estimated annual percentage change (EAPC) was calculated using the formula 100 * (e^β − 1), accompanied by a 95% confidence interval (95% CI). An upward trend in ASR was determined if both the EAPC and the lower limit of the 95% CI were positive, whereas a decline was noted if the EAPC and the upper limit of the 95% CI were negative. Stability in ASR was inferred if neither condition was met (19, 20). The correlation between ASR and the Socio-demographic Index (SDI) was investigated using a Gaussian process regression model with Loess smoothing, assessed via Spearman rank correlation tests (19, 21). Decomposition analysis was performed to assess the effects of aging, population growth, and epidemiological changes on DALY variations from 1990 to 2021, as elaborated in earlier studies (22).

Moreover, the ARIMA (Autoregressive Integrated Moving Average) model was utilized to examine the effects of air pollution on diabetes mellitus trends and to project these trends globally, regionally, and nationally from 2020 to 2050. In the ARIMA model configuration (p, d, q), ‘AR’ indicates the autoregressive part with p representing the number of lag observations included in the model; ‘MA’ denotes the moving average segment with q indicating the number of lag forecast errors; and d specifies the degree of differencing required for data stabilization (23). The model’s selection was refined based on the Akaike Information Criterion (AIC) and the Bayesian Information Criterion (BIC).

Uncertainty intervals (UI) of 95% were determined for all calculated figures (24). The rates were presented per 100,000 population. Data management, analysis, and visualization tasks were executed using R software, version 4.3.2 (25–29).

## 3 Result

### 3.1 Spatiotemporal patterns of diabetes burden attributable to air pollution

In 2021, air pollution was responsible for roughly 281.91 thousand deaths and 12.90 million disability-adjusted life years (DALYs) related to diabetes mellitus, featuring an age-standardized mortality rate (ASMR) of 3.3234 (95% UI, 1.9549–4.6634) and an age-standardized DALY rate (ASDR) of 148.9167 (95% UI, 86.5013–224.9116) per 100,000 individuals. Over the past three decades, there has been a marked rise in the diabetes burden attributable to air pollution (**Table 1**, **Supplementary Table S1**).

**Figure 1:**
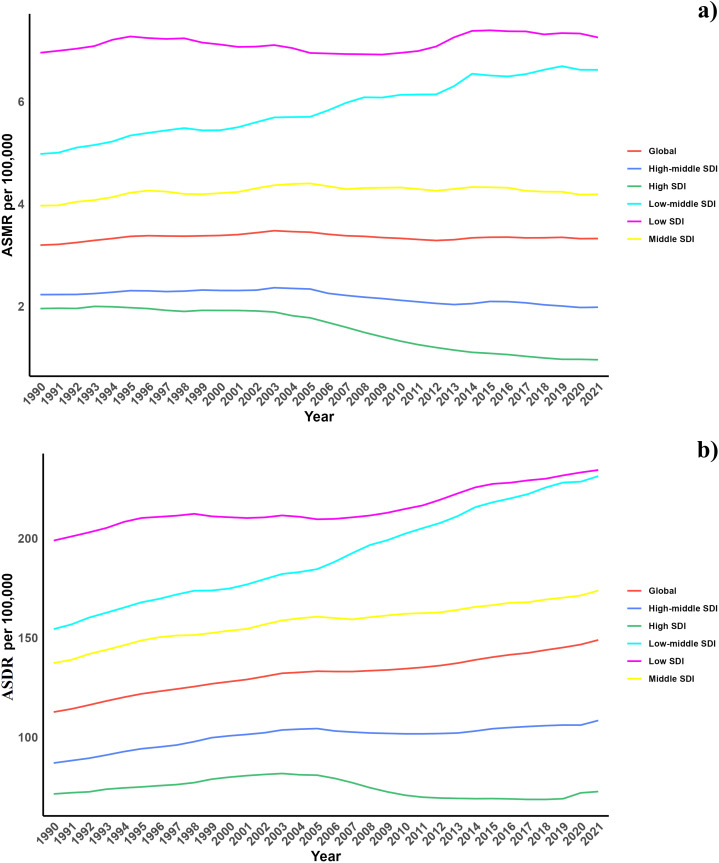
**(a)** Temporal trends of ASMR of diabetes mellitus attributable to air pollution from 1990 to 2021 in different SDI regions. **(b)** Temporal trends of ASDR of diabetes mellitus attributable to air pollution from 1990 to 2021 in different SDI regions.

In terms of the Socio-demographic Index (SDI), while high SDI and high-middle SDI regions have experienced a decrease in the diabetes burden due to air pollution, other regions have observed an increase. Additionally, an increase in the overall burden was noted across all SDI regions (**Table 1**, **Supplementary Table S1**, **Figure 1**

**Table 1:** Global and regional deaths and DALYs of diabetes mellitus attributable to air pollution in 1990 and 2021 in 27 global regions.

Location	Deaths Number in 1990	Deaths Number in 2021	ASMR in 2021	DALY Number in 1990	DALY Number in 2021	ASDR in 2021
**Global**	117055.1981 (165962.1725, 69992.0516)	281908.8380 (395527.8182, 165678.1738)	3.3234 (4.6634, 1.9549)	4568390.0872 (6604453.5510, 2676365.0738)	12904493.5493 (19485253.9069, 7501414.2293)	148.9167 (224.9116, 86.5013)
**Region**						
East Asia	14727.5217 (20911.7647, 8881.8319)	37751.9552 (55167.0425, 23300.1518)	1.8276 (2.6631, 1.1295)	824439.0782 (1245827.7639, 479151.7348)	2387936.4267 (3684560.5727, 1386920.5967)	112.2851 (172.9835, 64.9136)
Southeast Asia	12791.3861 (18293.7769, 7899.1202)	36436.1704 (51043.5376, 22147.5947)	6.1075 (8.5699, 3.7109)	459576.2404 (668999.6680, 277287.7965)	1476712.3687 (2172697.5502, 874644.3192)	220.6117 (324.8253, 130.7635)
Oceania	492.4595 (745.7360, 291.5631)	1286.6016 (1966.6874, 706.3905)	19.0845 (29.0002, 10.4003)	17177.2223 (25830.7760, 10427.4353)	52693.2245 (79851.8979, 30148.2945)	621.3560 (945.7589, 355.1970)
Central Asia	746.4653 (1117.9430, 418.5825)	2488.6182 (3591.7984, 1462.0006)	3.0928 (4.4446, 1.8212)	35798.5655 (56922.9462, 19624.6453)	138518.8492 (207337.2660, 81459.3424)	155.8100 (233.2900, 91.5154)
Central Europe	3489.8882 (4995.3647, 2124.1619)	5438.4075 (7873.7798, 3135.5907)	2.3089 (3.3464, 1.3302)	159701.0535 (240033.3917, 87915.7510)	255097.4515 (395129.1260, 143860.4285)	120.2278 (186.5299, 67.3995)
Eastern Europe	1897.4284 (2748.8987, 1052.1553)	5028.5939 (8058.7657, 2530.9493)	1.3850 (2.2185, 0.6970)	125712.8564 (195719.1721, 67706.9107)	227548.2176 (365162.8899, 112777.5481)	66.4575 (106.6777, 32.5307)
High-income Asia Pacific	2581.6019 (4063.8218, 892.7968)	3441.3828 (4974.9071, 1989.7310)	0.6391 (0.9257, 0.3723)	135720.6213 (235118.5005, 44485.9463)	352888.8077 (571645.6177, 186920.1218)	96.6851 (157.4390, 50.3967)
Australasia	162.9260 (411.7248, 6.3782)	436.2037 (721.2144, 217.3460)	0.7295 (1.2000, 0.3631)	5404.8024 (14309.8875, 184.7536)	18748.2949 (32384.6249, 8877.3381)	36.7258 (63.6792, 17.2853)
Western Europe	14159.5754 (21006.8980, 7611.4867)	9966.1210 (14953.0625, 5532.3714)	0.8670 (1.3018, 0.4886)	394566.4027 (594536.6140, 212282.0242)	413144.0819 (671273.1168, 213306.2754)	49.8410 (82.8157, 25.3066)
Southern Latin America	1550.7860 (2406.7956, 829.1138)	1912.1466 (2911.1477, 1041.4349)	2.1255 (3.2410, 1.1598)	49491.6721 (77674.6896, 25784.0562)	93484.2589 (152262.4287, 50637.3706)	108.9616 (177.1198, 58.9048)
High-income North America	6881.3038 (11016.2108, 2741.3070)	4475.0174 (8056.9680, 1817.8266)	0.6633 (1.1933, 0.2694)	246151.2045 (402247.6676, 96027.9795)	301055.3128 (562699.8736, 117266.5579)	49.3951 (92.3140, 19.2403)
Caribbean	1695.2934 (2534.4880, 890.8865)	3069.0994 (4680.7284, 1750.6287)	5.6684 (8.6432, 3.2373)	58820.3235 (90973.0032, 30474.7866)	140828.8269 (218925.6747, 77559.0644)	262.9395 (409.4020, 144.7345)
Andean Latin America	782.2747 (1112.6003, 466.7733)	2565.3712 (3724.1001, 1459.6133)	4.4561 (6.4632, 2.5362)	28148.1111 (40902.9454, 16680.9748)	105561.3220 (158662.8863, 59919.8403)	176.2803 (264.6323, 100.1207)
Central Latin America	7081.5374 (10037.6700, 4398.2229)	18235.8409 (27256.0315, 10856.8972)	7.4408 (11.1154, 4.4297)	270263.3370 (395222.0916, 160774.5611)	719122.7417 (1087740.2597, 413501.1583)	280.3022 (423.2255, 161.6501)
Tropical Latin America	4139.9958 (6413.2387, 1915.8542)	8304.6454 (13268.1751, 4179.8888)	3.3167 (5.3095, 1.6702)	158441.0346 (250641.1484, 76699.0603)	338172.2017 (567838.6979, 173090.7017)	130.2282 (218.3913, 66.6377)
North Africa and Middle East	6552.4805 (9291.4332, 3949.1182)	22388.3259 (31732.6698, 13338.5734)	5.7063 (8.0298, 3.3953)	254835.1041 (372106.4090, 152680.9399)	1230289.9345 (1852439.9446, 720894.9538)	255.6159 (383.5728, 150.0744)
South Asia	22522.9288 (31817.3960, 13753.1357)	82661.2192 (117963.7517, 48643.2485)	6.4430 (9.1718, 3.8023)	874625.7825 (1270423.1982, 521885.8734)	3346802.8601 (4919034.9482, 1987992.7285)	220.4004 (321.6524, 130.8132)
Central Sub-Saharan Africa	1929.6030 (2815.4227, 1174.9889)	4639.7403 (6893.6914, 2693.5834)	10.2495 (14.9176, 6.0600)	64505.7477 (93396.5765, 39294.4031)	188123.2643 (280297.8027, 108997.9301)	313.9963 (463.5407, 181.9338)
Eastern Sub-Saharan Africa	6022.3026 (8679.7887, 3625.4875)	11640.4778 (16705.6779, 6745.2431)	8.2715 (11.7470, 4.7620)	185740.6114 (268457.1093, 109893.0197)	400931.7369 (578903.7322, 234032.2020)	226.2891 (324.8920, 131.4685)
Southern Sub-Saharan Africa	1997.5890 (2844.1628, 1217.2715)	7016.5256 (10225.8838, 4254.8601)	13.7259 (19.9942, 8.2911)	62084.9723 (89645.7421, 37454.4042)	221361.0138 (320787.4753, 134034.4476)	379.2860 (548.2145, 229.6119)
Western Sub-Saharan Africa	4849.8507 (6998.6712, 2934.7707)	12726.3741 (18335.8386, 7562.4411)	7.9127 (11.4000, 4.7694)	157185.3437 (229960.0314, 92654.1773)	495472.3529 (721787.9051, 291548.7828)	239.8894 (345.9861, 142.4705)
**SDI**						
High-middle SDI	20173.3690 (28802.9125, 12103.6235)	38951.2947 (56060.0623, 23560.4730)	1.9804 (2.8529, 1.1989)	871726.0750 (1311374.9800, 510336.5721)	2080648.5439 (3196593.9305, 1180843.7641)	108.4117 (167.7089, 61.6624)
High SDI	21821.4335 (32583.3718, 11950.2465)	21695.3443 (32950.6270, 12189.9508)	0.9546 (1.4458, 0.5400)	776220.5040 (1194573.3961, 411469.9721)	1339270.3990 (2192458.4179, 698466.1025)	72.6476 (119.2426, 37.7252)
Low-middle SDI	26232.9461 (37438.1812, 15932.0077)	84425.6851 (118940.2042, 50251.1953)	6.6191 (9.3593, 3.9587)	972904.8319 (1418692.0810, 589820.0050)	3433414.7602 (5056634.7496, 2033882.2760)	231.3510 (338.4636, 136.3710)
Low SDI	13613.2478 (19515.6433, 8247.1861)	31013.1188 (44159.4322, 18332.8679)	7.2531 (10.2497, 4.2973)	465812.1450 (673243.1667, 281964.2837)	1263109.0667 (1828692.8957, 753685.6540)	234.4284 (342.1238, 139.5443)
Middle SDI	35031.1495 (49607.8278, 20775.8184)	105491.6752 (149953.5829, 62747.3338)	4.1866 (5.9240, 2.4868)	1474810.6300 (2128310.7871, 883136.3560)	4773270.5045 (7165713.8618, 2774297.9443)	173.7644 (260.5925, 101.1816)

Region-wise, East Asia, Southeast Asia, and South Asia registered the highest impact with the most deaths and DALYs, whereas Oceania, Central Asia, and Australasia recorded the lowest. Conversely, in terms of ASMR and ASDR, Oceania, Central Sub-Saharan Africa, and Southern Sub-Saharan Africa demonstrated the highest burden (**Table 1**).

**Figure 2:**
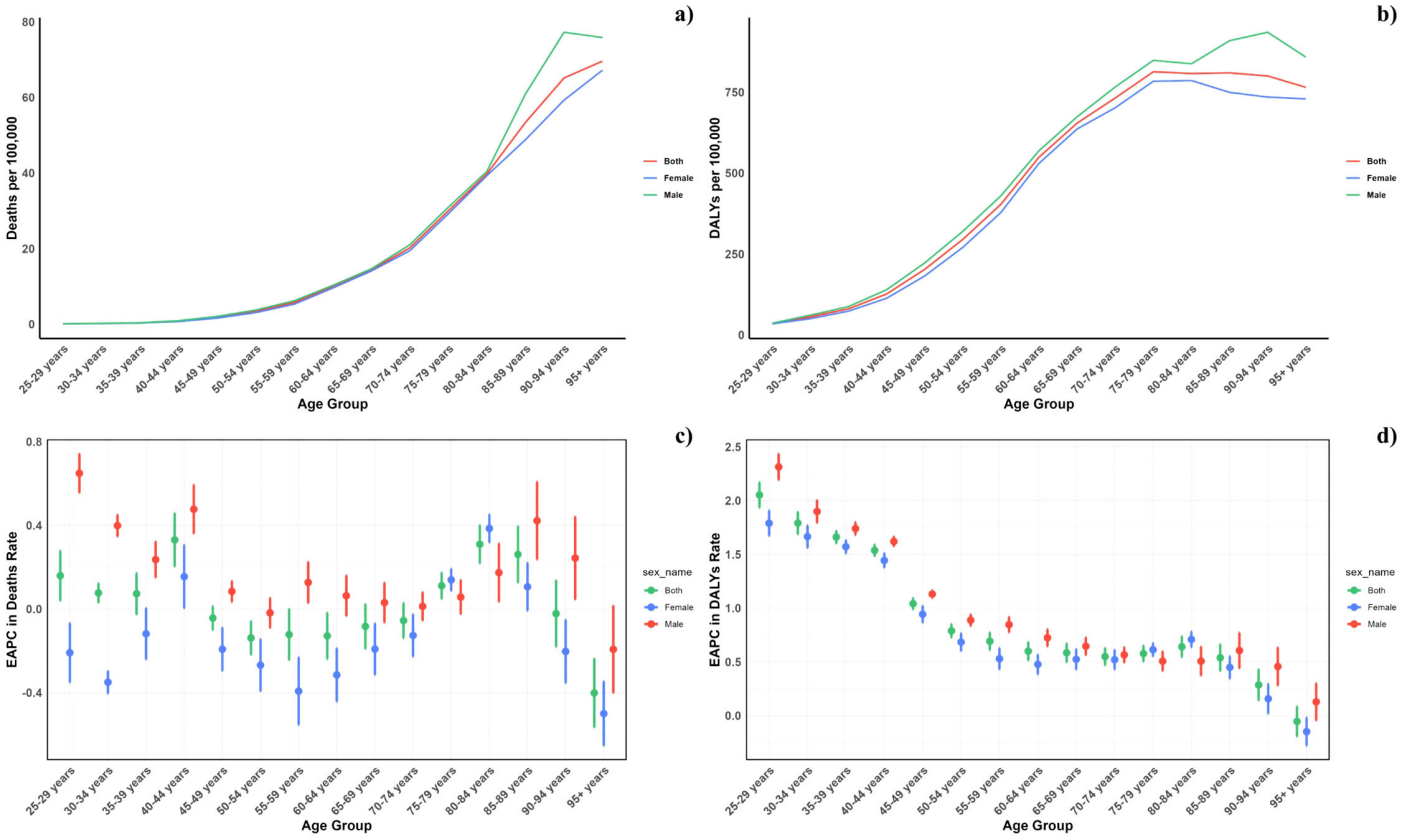
Age–specific rates of global deaths **(a)** and DALYs **(b)** of diabetes mellitus attributable to air pollution, by sex, in 2021 and the corresponding EAPC of global deaths **(c)** and DALYs **(d)** from 1990 to 2021.

**Figure 3:**
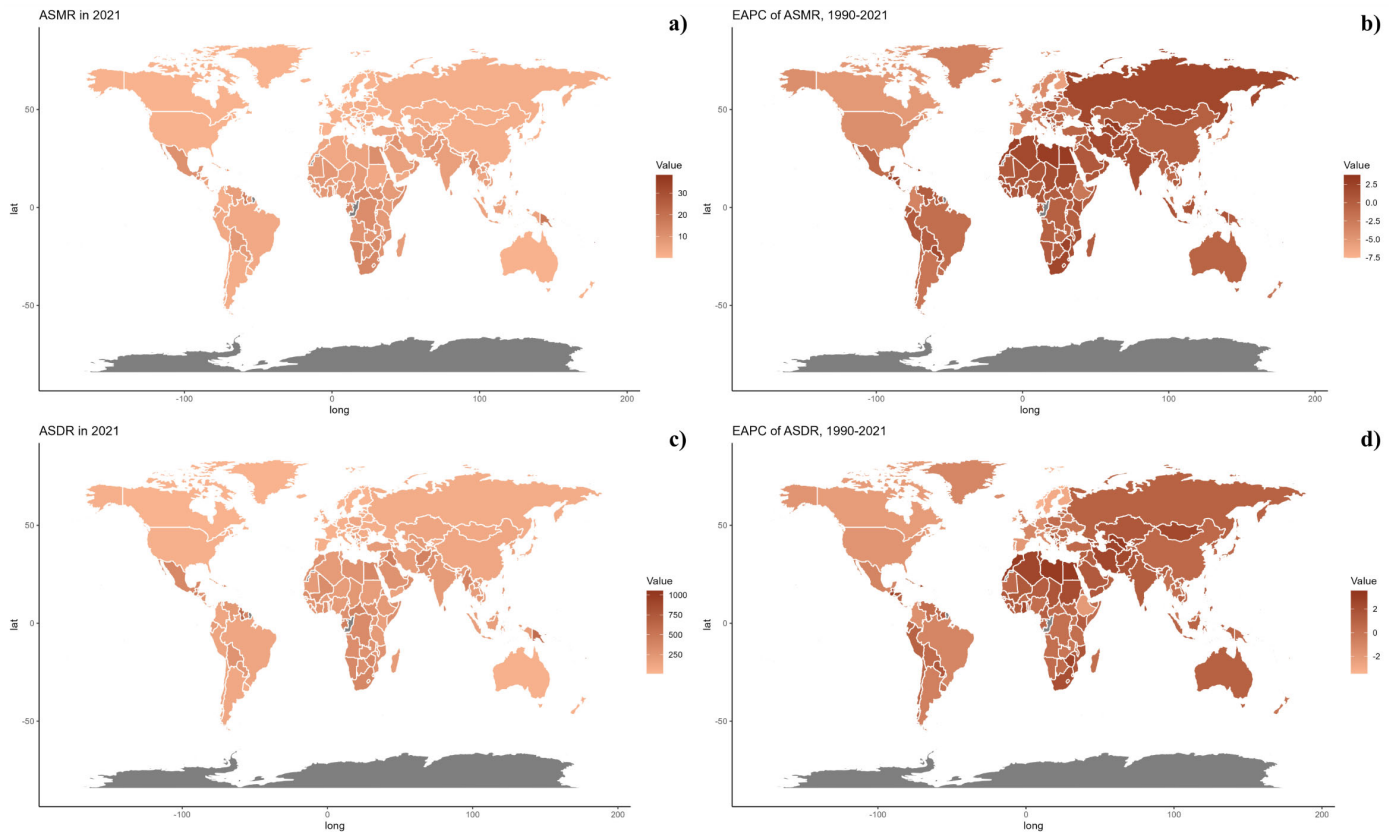
**(a)** Age-specific rates of global deaths of diabetes mellitus attributable to air pollution, by sex, in 2021. **(b)** Age-specific rates of global DALYs (b) of diabetes mellitus attributable to air pollution, by sex, in 2021. **(c)** Corresponding EAPC of global deaths from 1990 to 2021. **(d)** Corresponding EAPC of global DALYs (d) from 1990 to 2021.

At the national level, in 2021, there was significant variability in ASMR across countries, with populous nations like China and India showing the highest rates, reflecting major health challenges linked to environmental and public health issues (**Figure 2**). From 1990 to 2021, some countries, especially in Western Europe and North America, saw a decline in ASMR, indicating improvements in health metrics. However, countries such as Honduras and Libya experienced increases, primarily due to PM2.5 pollution and other environmental concerns, underscoring unique regional health challenges that differ from the global trend toward better health outcomes (**Table 1**, **Supplementary Table S1**, **Figure 3**). The geographical and chronological trends of ASDR mirrored those of ASMR.

### 3.2 Age and sex patterns

**Figure 4:**
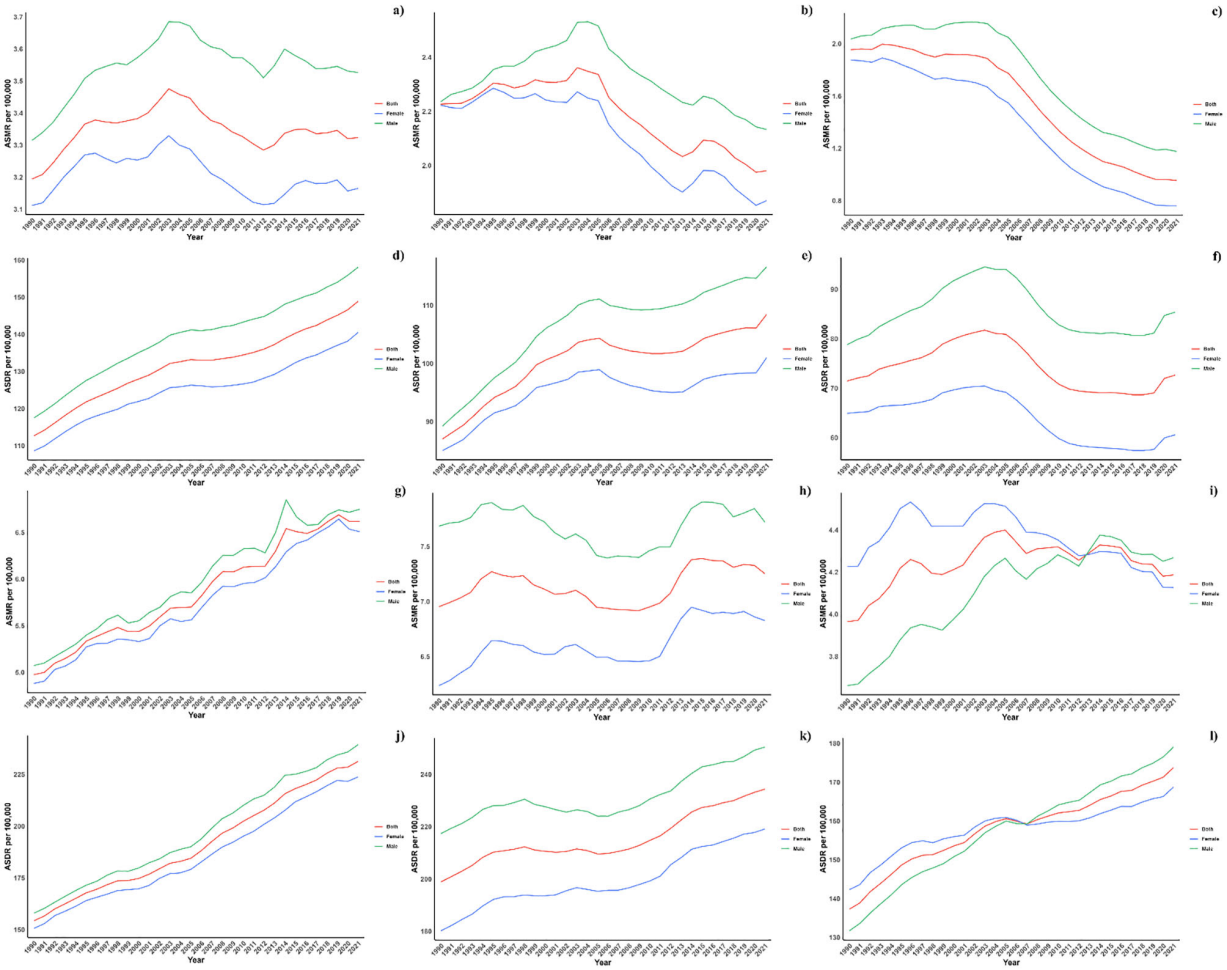
**(a)** ASMR in diabetes mellitus Burden Attributable to Air Pollution across Global Regions. **(b)** ASMR in diabetes mellitus Burden Attributable to Air Pollution across High-middle SDI Regions. **(c)** ASMR in diabetes mellitus Burden Attributable to Air Pollution across High SDI Regions. **(d)** ASMR in diabetes mellitus Burden Attributable to Air Pollution across Low-middle SDI Regions. **(e)** ASMR in diabetes mellitus Burden Attributable to Air Pollution across Low SDI Regions. **(f)** ASMR in diabetes mellitus Burden Attributable to Air Pollution across Middle SDI Regions. **(g)** ASDR in diabetes mellitus Burden Attributable to Air Pollution across Global Regions. **(h)** ASDR in diabetes mellitus Burden Attributable to Air Pollution across High-middle SDI Regions. **(i)** ASDR in diabetes mellitus Burden Attributable to Air Pollution across High SDI Regions. **(j)** ASDR in diabetes mellitus Burden Attributable to Air Pollution across Low-middle SDI Regions. **(k)** ASDR in diabetes mellitus Burden Attributable to Air Pollution across Low SDI Regions. **(l)** ASDR in diabetes mellitus Burden Attributable to Air Pollution across Middle SDI Regions.

**Figure 2** illustrates the age-specific global mortality and DALY rates for diabetes mellitus in 2021, along with changes from 1990. These rates reveal a J-shaped pattern, indicating an increase in mortality and DALYs among individuals below 60, with a significant surge observed in the 60+ age groups. Across all age brackets, males demonstrated consistently higher mortality rates from air pollution than females. Similarly, the DALY rates associated with air pollution were elevated for all age groups. In every SDI region, males exhibited higher mortality and DALY rates, with this gender difference being consistent across the regions (**Figure 4**).

### 3.3 Association with the socio-demographic index

**Figure 5:**
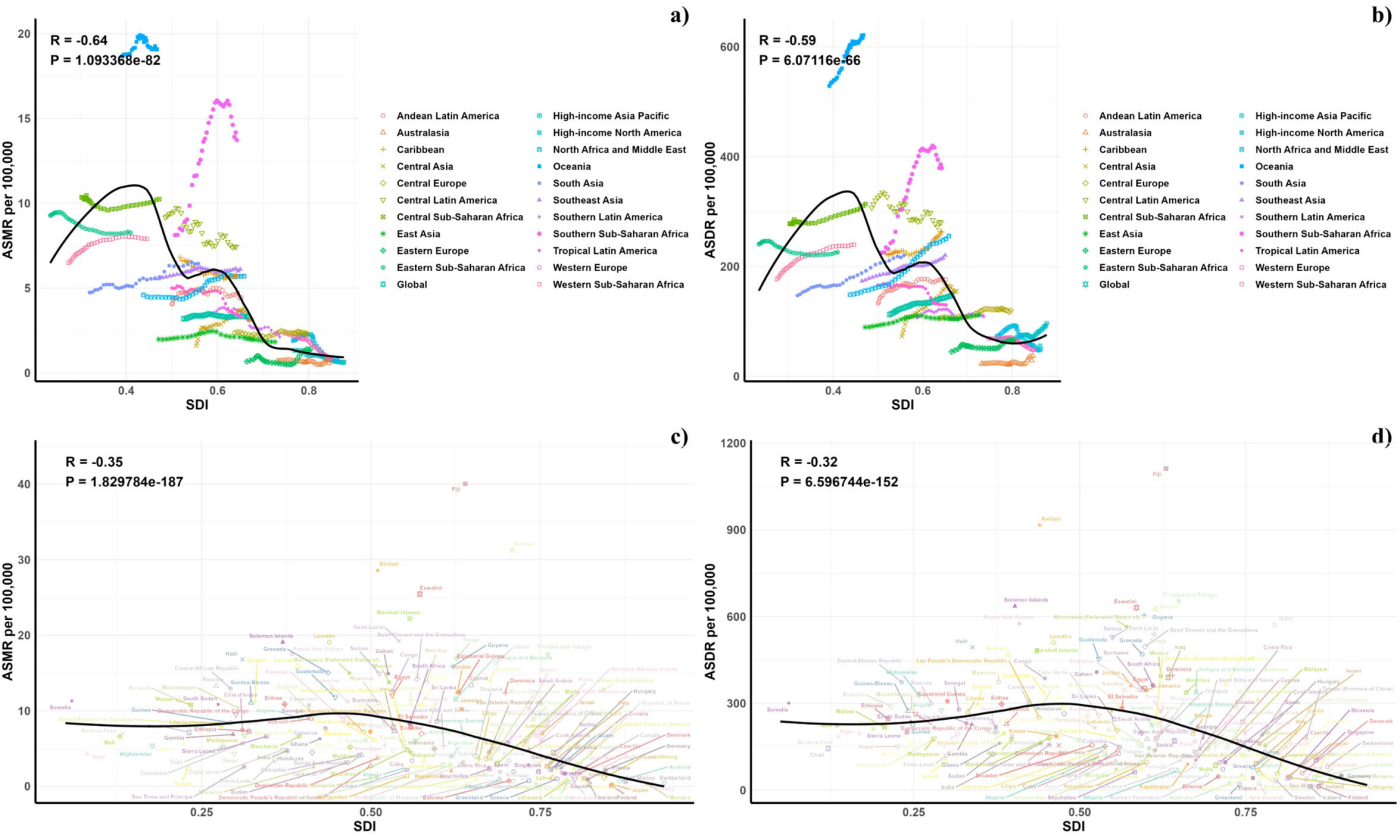
Correlations between ASMR **(a, c)** and ASDR **(b, d)** of diabetes mellitus attributable to air pollution and SDI at the regional level **(a, b)** and the national level **(c, d)**.

**Figure 5** presents a comparison of the observed and forecasted age-standardized DALY (ASDR) and mortality rates (ASMR) due to air pollution, juxtaposed with Socio-Demographic Index (SDI) values at both regional and national levels from 1990 to 2021. A negative correlation was observed between ASDR and SDI, indicating a reduction in burden with increasing SDI. Areas such as Oceania, southern sub-Saharan Africa, and central Latin America recorded higher ASDR than anticipated during this timeframe. Moreover, the observed ASDR figures were higher than projected in western sub-Saharan Africa and Australia. The trends for observed versus projected ASMR based on SDI at regional levels were consistent with the ASDR results. **Figure 5** also illustrates the observed versus projected ASDR and ASMR at the national level for 2021, revealing a similar inverse relationship between these rates and SDI, both regionally and nationally.

### 3.4 Forecasts for the mortality, DALYs rate, ASMR and ASDR of diabetes burden attributable to air pollution in global (2022–2050)

**Figure 6:**
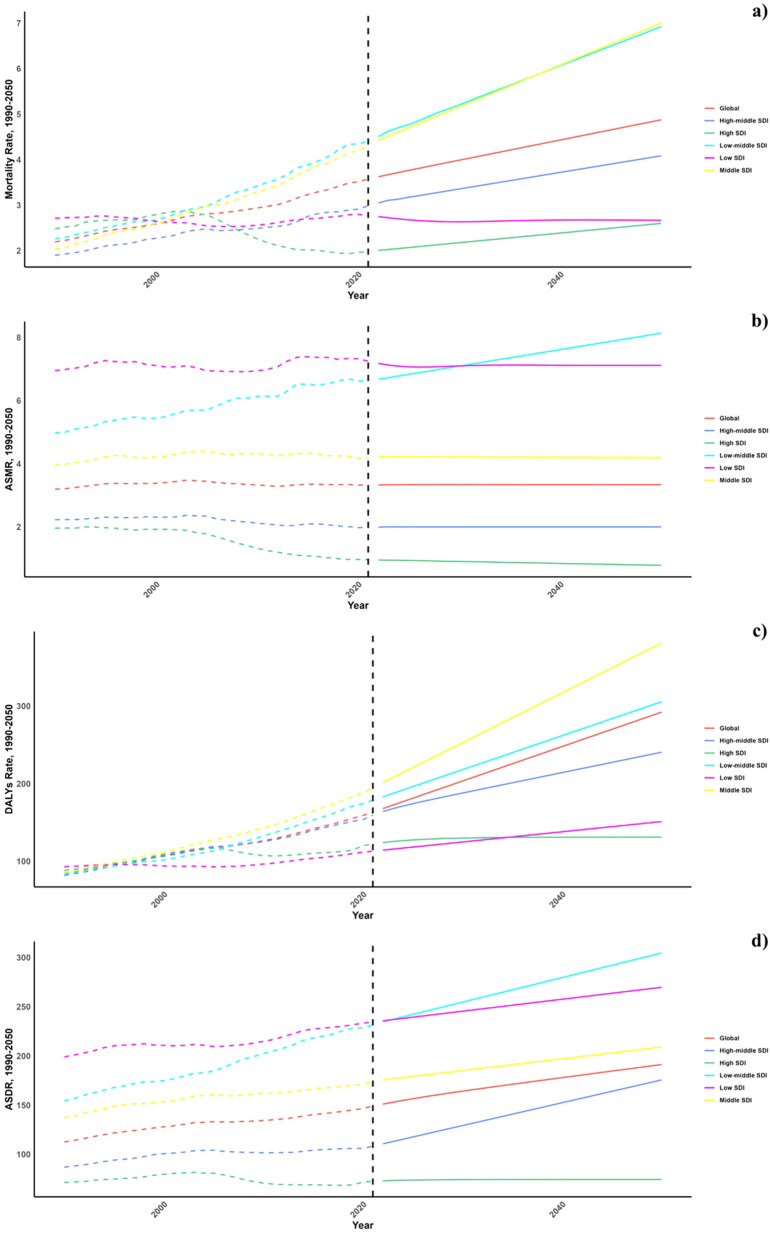
Estimated Trends of Mortality Rate **(a)**, DALYs Rate **(b)**, ASMR **(c)** and ASDR **(d)**, 1990–2050 at the Regional Level.

**Figure 7:**
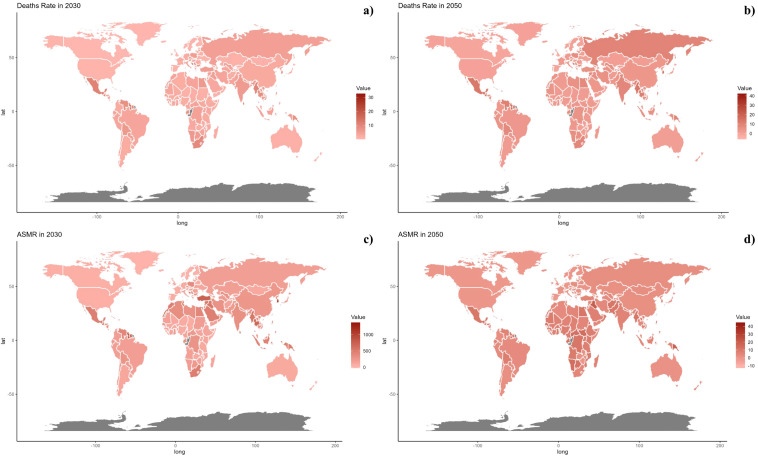
Estimated Trends of Mortality Rate **(a, b)** and ASMR **(c, d)** in 2030 **(a, c)** and 2050 **(b, d)** at the National Level.

**Figure 8:**
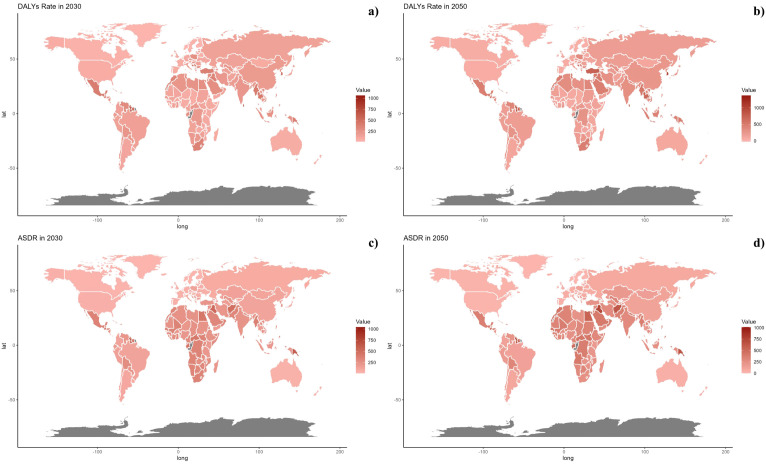
Estimated Trends of DALYs Rate **(a, b)** and ASDR **(c, d)** in 2030 **(a, c)** and 2050 **(b, d)** at the National Level.

Projections for mortality rates, DALY rates, ASMR, and ASDR related to CVDs due to air pollution are detailed in **Figures 6**–**8**. Regionally, the mortality rate is expected to decline only in the low SDI region. However, age-standardized assessments indicate that the low-middle SDI region is likely to see an increase. For DALY rates and ASDR, all SDI regions, with the exception of the high SDI, are anticipated to experience significant increases in the future (**Figure 6**).

On a national scale, the patterns are expected to stay stable in both 2030 and 2050. However, the projected death rates and ASMR from diabetes mellitus due to air pollution are notably higher in Russia, Mexico, and most African countries compared to others for both 2030 and 2050. In terms of ASDR, nations in South Asia and Africa are expected to witness more significant increases than other countries (**Figures 7**, **8**).

## 4 Discussion

This study examined the diabetes burden worldwide resulting from air pollution between 1990 and 2021, identifying a significant upward trend during this timeframe. The most impacted demographic included individuals over the age of 60, with males showing higher rates. Projections indicate that while mortality rates might decrease in regions with a low Social Development Index (SDI), standardized mortality rates are expected to rise in lower-middle SDI areas. Moreover, diabetes mortality and age-standardized death rates associated with air pollution are projected to remain elevated in Russia, Mexico, and many African nations, with a notably sharper increase in age-standardized mortality rates expected in South Asia and Africa compared to other regions. To our knowledge, this research is the first to thoroughly assess diabetes burden from air pollution.

In 2017, PM2.5 exposure contributed to 4.58 million deaths and 142.52 million DALYs worldwide (30). While age-standardized rates (ASRs) of deaths and DALYs saw a decline, the decrease was modest on a global scale, with certain regions like Latin America, Africa, South Asia, the Middle East, and Oceania experiencing an increase in the absolute burden as the global population rose from 5.3 billion in 1990 to 7.6 billion in 2017. With rapid industrialization and urbanization, the health impact of ambient PM2.5 in low- and middle-income areas has become increasingly apparent. Concurrently, Cookstove Intervention Programs expanded, reducing household PM2.5 burdens in developing nations, though only 60% of the global population had access to clean fuels (31–34). Significantly, five Asian nations (India, China, Pakistan, Indonesia, and Bangladesh) represented the highest burdens, accounting for over 50% of all deaths and DALYs related to PM2.5.

According to the World Health Organization, as economic conditions and sanitation improved, non-communicable diseases (NCDs) represented over 60% of the global disease burden and contributed to more than 70% of deaths in 2017 (35). Major diseases linked to PM2.5 were among the top 10 causes of death worldwide in 2016 (36). The leading three conditions for PM2.5-associated DALYs were ischemic heart disease (IHD), cerebrovascular disease, and lower respiratory infections (LRI), followed by chronic obstructive pulmonary disease (COPD), diabetes, and lung cancer, all of which ranked in the top 20 causes of global DALYs (37). Our findings indicated an increase in deaths and DALYs from PM2.5-related NCDs particularly among those over 70, while the 14-69 age group largely contributed to DALYs in conditions like stroke, IHD, and diabetes, posing significant challenges for public health and socio-economic development by impacting the labor force.

Studies have shown that economic growth has often led to substantial environmental pollution which adversely affects glucose metabolism, promoting insulin resistance and Type 2 Diabetes Mellitus (T2DM, 39). Meta-analyses have established a positive association between significant air pollutants (like PM2.5, PM10, NO2, and NOX) and T2DM (38). From 2000 to 2015, PM2.5 levels escalated from 31.2 μg/m3 to 97.0 μg/m3 in 15 Chinese provinces and regions (39). Additionally, research has linked a 10 μg/m3 increase in PM2.5 exposure to a 10% heightened risk of developing T2DM (40). Consequently, there is an urgent need to address the issue of diabetes mellitus caused by air pollution.

Furthermore, our studies have shown that East Asia, Southeast Asia, and South Asia bear the greatest burden, with the highest numbers of deaths and DALYs, highlighting that densely populated and rapidly industrializing countries like India and China face disproportionately high disease burdens from PM2.5, exacerbated by significant rural-urban economic disparities and uneven public health resources. Between 2000 and 2010, health welfare costs and losses in labor productivity due to air pollution accounted for 6.5% of China’s GDP annually, and 8.5% of India’s GDP in 2013, while Bangladesh incurs around $6.5 billion annually in related costs. In response, the Chinese government implemented the Air Pollution Prevention and Control Action Plan, revised the Law on the Prevention and Control of Atmospheric Pollution, and took steps such as switching to natural gas for heating, regulating power plants, and limiting vehicle emissions (41–43). Similarly, the Indian government initiated the National Clean Air Program aimed at enhancing air quality monitoring, boosting pollution control capabilities, and reducing PM2.5 and PM10 levels by 20–30% by 2024. These proactive measures toward eco–friendly development are expected to mitigate public health losses from PM2.5, enhance economic dynamism through improved labor productivity and energy efficiency, and assist these countries in breaking out of detrimental cycles toward sustainable development.

Air pollution, especially PM2.5, ozone (O₃), and nitrogen oxides (NOx), can reduce vitamin D synthesis by blocking UVB radiation necessary for its production in the skin. This effect is particularly pronounced in highly polluted urban areas, where low UV exposure contributes to higher rates of vitamin D deficiency. Additionally, pollution–induced inflammation and oxidative stress can impair insulin sensitivity, increasing the risk of diabetes. Both reduced vitamin D levels and increased systemic inflammation from air pollutants may synergistically elevate the risk of type 2 diabetes, highlighting the need for effective environmental and health interventions (44).

Building on our findings, actionable policy recommendations are essential to mitigate the diabetes burden attributable to air pollution. Governments should prioritize stricter air quality regulations and promote cleaner energy alternatives to reduce particulate matter exposure, particularly in high–risk regions. Integrating air pollution mitigation strategies with existing public health programs targeting diabetes prevention could amplify their impact. Additionally, improving urban planning to decrease air pollution in densely populated areas and raising public awareness about the health risks associated with air pollution are critical. Future policies should also emphasize tailored interventions for vulnerable populations, such as the elderly and individuals in lower SDI regions.

In the context of our study findings, the recent public health measures and policy developments in countries like China and India provide insightful examples. China’s Air Pollution Prevention and Control Action Plan and India’s National Clean Air Program (NCAP) aim to significantly reduce PM2.5 and PM10 levels. These initiatives demonstrate a proactive approach to mitigating air pollution and its associated health impacts, including diabetes mellitus. By incorporating these national efforts into our discussion, we can better illustrate the potential for public health policies to influence and possibly reduce the burden of diseases exacerbated by environmental pollutants (45–47).

Our research encounters several notable constraints. Primarily, there is a deficiency of primary data from less developed areas, particularly in Sub–Saharan Africa, where assessments are predominantly based on mathematical modeling, leading to wide ranges of uncertainty. Additionally, air pollution comprises a complex mixture of various components, each possessing unique physicochemical properties and toxic effects that vary significantly across different regions and seasons.

## 5 Conclusion

This study offers an in–depth analysis of the global burden of diabetes mellitus attributed to air pollution from 1990 to 2021, observing a significant increase over the years. Individuals aged over 60 were the most impacted, with men facing a greater burden. Looking ahead, only regions with a low Socio–Demographic Index (SDI) are expected to see a decrease in mortality rates, whereas those in lower–middle SDI regions will likely witness an increase in standardized mortality rates. On a national scale, countries such as Russia, Mexico, and most of Africa are anticipated to continue experiencing elevated diabetes mortality rates and ASMR due to air pollution up to 2030 and 2050. Both South Asia and Africa are projected to encounter substantial rises in ASDR compared to other areas.

This underscores the urgent necessity for policymakers to devise and refine preventive measures tailored to specific populations to reduce the diabetes burden associated with air pollution in the future.

## Data Availability

The raw data supporting the conclusions of this article will be made available by the authors, without undue reservation.
