# Supplementary material for: Diabetes burden attributable to air pollution from 1990~2021 and the future trends: a population-based study
Source: Front Endocrinol (Lausanne). 2025 Apr 8;16:1475822. doi: 10.3389/fendo.2025.1475822 (PMC12011618; doi:10.3389/fendo.2025.1475822)
Supplement: Supplementary file 1 [file Table1.docx]

Table S1 Global and regional deaths and DALYs of diabetes mellitus attributable to air pollution in 1990 and 2021 in 204 nations

| Location | Deaths Number in 1990 | Deaths Number in 2021 | ASMR in 2021 | DALY Number in 1990 | DALY Number in 2021 | ASDR in 2021 |
| --- | --- | --- | --- | --- | --- | --- |
| China | 13430.6175 (19127.1489, 8094.5686) | 35146.3560 (51416.4575, 21614.2870) | 80.2729 (101.6316, 61.1427) | 777143.9081 (1176467.6908, 451502.8470) | 2274206.9136 (3507096.0096, 1317236.0853) | 1432.8498 (1826.6884, 1100.8764) |
| Democratic People's Republic of Korea | 384.6171 (594.2284, 223.6029) | 892.1901 (1330.5222, 531.3048) | 146.4569 (184.1631, 112.2008) | 17010.9417 (25340.5675, 10054.0950) | 48303.4401 (73087.5428, 28449.5455) | 3171.1241 (4011.9804, 2438.2198) |
| Taiwan (Province of China) | 912.2871 (1346.1363, 484.2539) | 1713.4091 (2465.3081, 1017.8106) | 9.8220 (12.3850, 7.5678) | 30284.2284 (45717.4615, 16561.4194) | 65426.0730 (99131.7998, 37379.9524) | 222.9873 (277.2028, 175.6988) |
| Cambodia | 237.7678 (356.9093, 145.4987) | 711.5777 (1106.3786, 402.8895) | 130.3558 (157.9727, 100.0271) | 8068.4375 (12041.1783, 4860.7305) | 29400.4904 (44152.3797, 17036.1431) | 2591.6896 (3201.3333, 1975.3013) |
| Indonesia | 3510.8413 (5143.1781, 2141.0820) | 10692.4299 (15429.7594, 6328.5134) | 83.0524 (112.1290, 57.5077) | 139047.7346 (204523.1321, 83963.0926) | 461333.9857 (674144.0702, 273829.3112) | 1731.3833 (2344.7297, 1193.3650) |
| Lao People's Democratic Republic | 142.2081 (217.0668, 82.7630) | 298.3906 (450.4579, 165.7704) | 151.8194 (194.4992, 106.4069) | 4893.2541 (7434.9657, 2935.2960) | 13082.0599 (19793.1809, 7719.6797) | 3195.0592 (4157.2303, 2191.0091) |
| Malaysia | 429.9830 (646.1306, 241.9927) | 848.2774 (1273.5839, 505.4227) | 38.6500 (53.2690, 26.1973) | 17226.8435 (25929.4293, 9557.7489) | 50756.0210 (79628.3010, 28636.4648) | 830.5487 (1144.1633, 559.6716) |
| Maldives | 5.3297 (7.8178, 3.1469) | 6.9781 (11.5340, 3.5353) | 17.4665 (27.2985, 9.7479) | 194.5292 (284.1998, 115.6422) | 401.8434 (677.8815, 202.4165) | 331.3023 (519.0493, 185.3105) |
| Myanmar | 2501.8522 (3729.6393, 1461.0676) | 5004.8820 (7378.5746, 2968.2987) | 136.7202 (176.0094, 102.3439) | 84326.6854 (122772.6937, 50992.9985) | 193252.4925 (280996.7415, 113392.1030) | 2860.3659 (3678.8292, 2126.4424) |
| Philippines | 1455.1158 (2064.2255, 874.5881) | 5754.7408 (8627.1285, 3522.5075) | 84.4575 (106.0281, 61.5122) | 57158.1387 (82201.8619, 34030.8234) | 213439.1265 (314653.0917, 126915.0872) | 1892.2250 (2396.4128, 1382.3514) |
| Sri Lanka | 672.1746 (963.4738, 400.5300) | 2312.9629 (3549.7841, 1216.4474) | 53.9805 (89.2746, 27.7445) | 24354.3838 (35683.5636, 14417.0604) | 99759.1474 (151264.6590, 58445.9610) | 1029.6348 (1721.7724, 539.1578) |
| Thailand | 1553.3545 (2273.6886, 931.8224) | 4788.6254 (7008.6335, 2756.5199) | 28.7756 (38.8778, 20.6126) | 57012.2856 (82502.9459, 33994.8380) | 207415.3831 (304983.1381, 121132.2105) | 667.7611 (895.8098, 481.4765) |
| Timor-Leste | 9.0776 (13.6943, 5.2550) | 34.4760 (54.7645, 19.3992) | 129.3173 (174.0153, 80.7545) | 356.5378 (525.6559, 210.2154) | 1721.0719 (2645.3218, 998.5528) | 2647.6512 (3542.3054, 1654.2052) |
| Viet Nam | 2221.2610 (3299.5327, 1339.4071) | 5757.4509 (8518.8433, 3271.6355) | 79.5006 (101.3385, 56.5900) | 65003.4709 (95600.4527, 38431.5213) | 198205.9672 (299904.2710, 115292.6403) | 1554.7047 (2004.6969, 1105.3314) |
| Fiji | 100.5592 (152.8594, 54.9638) | 261.5231 (435.6202, 101.0184) | 73.7132 (128.7420, 24.1705) | 3298.5740 (4975.7164, 1818.8991) | 8504.4865 (14474.0252, 3393.4005) | 1619.6205 (2872.3999, 518.0555) |
| Kiribati | 9.3173 (13.4557, 5.5622) | 18.0868 (31.6058, 8.3727) | 122.3802 (158.4607, 91.9498) | 313.2129 (452.5515, 183.8739) | 669.6622 (1161.9040, 318.7663) | 2993.9114 (3950.3091, 2218.7149) |
| Marshall Islands | 2.2091 (3.8811, 0.8795) | 6.8778 (13.3763, 2.2811) | 111.6297 (162.9092, 75.4039) | 82.5607 (143.7791, 33.1960) | 306.4748 (583.9037, 113.4256) | 2634.5580 (3917.0275, 1771.2446) |
| Micronesia (Federated States of) | 6.7672 (10.3314, 3.6062) | 11.0437 (20.5847, 4.1130) | 113.0268 (165.3850, 71.7174) | 225.4832 (342.6139, 118.9285) | 447.1428 (810.9897, 171.5202) | 2668.1585 (3946.6863, 1703.6695) |
| Papua New Guinea | 287.2750 (450.5387, 164.5841) | 784.8120 (1203.6304, 452.5746) | 143.2119 (185.8379, 103.8893) | 10254.0034 (15884.6855, 5959.9494) | 34593.5129 (51905.9102, 20366.4298) | 3201.1770 (4192.3412, 2311.4941) |
| Samoa | 10.9114 (16.1740, 6.5048) | 21.2539 (33.8851, 11.4008) | 127.9249 (170.5715, 86.1713) | 379.5544 (562.5580, 222.3243) | 873.8740 (1400.4447, 466.1464) | 2838.5652 (3807.8747, 1892.1535) |
| Solomon Islands | 20.9305 (33.4463, 10.7882) | 70.9233 (106.9940, 40.3087) | 230.6303 (291.6115, 179.0788) | 724.2904 (1151.5514, 380.9902) | 2722.3962 (4108.1537, 1629.9088) | 5194.0651 (6679.0335, 4018.2458) |
| Tonga | 8.3544 (12.6041, 4.7387) | 13.2015 (21.4099, 5.9870) | 56.9549 (82.1380, 34.5176) | 273.2628 (411.9671, 153.6335) | 447.6961 (731.7715, 201.4183) | 1244.1775 (1826.0178, 757.5829) |
| Vanuatu | 7.8649 (12.9428, 4.3220) | 27.1020 (41.5782, 15.7824) | 216.3345 (258.1585, 172.8357) | 278.9888 (445.4973, 160.1918) | 1115.1681 (1681.6067, 658.7710) | 5054.5733 (6101.4687, 4006.8488) |
| Armenia | 84.6115 (124.8609, 45.4148) | 119.9863 (172.4814, 70.6864) | 77.5780 (102.9148, 53.3281) | 3458.8096 (5258.9638, 1834.2774) | 5891.2285 (8890.4474, 3412.2222) | 1427.1951 (1895.3706, 990.5066) |
| Azerbaijan | 93.7552 (148.0437, 49.2010) | 304.6012 (475.5728, 158.4229) | 83.8336 (128.9953, 45.3611) | 4081.6826 (6512.6116, 2127.7883) | 16182.0140 (24945.0706, 8844.9372) | 1529.8659 (2368.2234, 828.5039) |
| Georgia | 109.0590 (167.6549, 57.5895) | 175.6198 (258.2340, 98.3273) | 54.4661 (77.3068, 34.2346) | 4684.0406 (7546.5893, 2502.2717) | 8075.7749 (12617.1200, 4442.7623) | 1076.6945 (1559.1817, 676.2879) |
| Kazakhstan | 131.9581 (209.1494, 66.3830) | 240.2535 (359.1023, 136.9165) | 74.0623 (99.6375, 51.6289) | 8494.1357 (14257.0912, 4216.4763) | 22812.0374 (35825.7730, 12833.8046) | 1297.5877 (1743.5417, 902.0771) |
| Kyrgyzstan | 26.7665 (39.1039, 15.1910) | 68.1553 (97.3439, 41.5954) | 112.9039 (142.7662, 84.7654) | 1572.2928 (2474.0362, 873.9045) | 5362.1194 (8161.0467, 3005.3975) | 2125.1288 (2678.6234, 1608.4701) |
| Mongolia | 10.4221 (16.5888, 5.8142) | 36.2735 (55.8570, 21.0573) | 109.0725 (146.6512, 75.6026) | 556.7610 (842.9124, 323.2038) | 2707.4801 (4126.6199, 1549.2997) | 2104.1813 (2827.8766, 1466.6074) |
| Tajikistan | 63.7901 (92.9607, 38.2524) | 155.4162 (241.4678, 87.8714) | 136.6317 (170.9367, 105.0439) | 2569.2133 (3863.3472, 1569.8893) | 9171.4100 (13579.2077, 5263.3144) | 2538.2233 (3152.0105, 1964.9155) |
| Turkmenistan | 27.9615 (48.0360, 10.3206) | 144.6454 (230.8015, 78.7215) | 91.5037 (141.0180, 53.7148) | 1177.2713 (2042.0342, 413.5766) | 6581.1330 (10242.9284, 3691.9702) | 1842.2543 (2840.9290, 1076.6980) |
| Uzbekistan | 198.1412 (291.3821, 113.0179) | 1243.6670 (1768.1313, 746.6066) | 134.9665 (175.1373, 97.4554) | 9204.3586 (13832.7821, 5192.7826) | 61735.6518 (90962.5733, 37157.2595) | 2556.0783 (3302.7895, 1869.3574) |
| Albania | 16.9504 (25.4028, 10.2560) | 34.3253 (54.3237, 18.7293) | 54.7468 (81.7546, 34.6149) | 1229.7503 (1898.0139, 692.5130) | 2792.9259 (4535.2126, 1512.8892) | 857.1307 (1288.2704, 543.7697) |
| Bosnia and Herzegovina | 106.0193 (150.1332, 62.3496) | 378.0596 (570.2195, 223.2344) | 69.2235 (93.9618, 48.7326) | 4909.7299 (7360.3094, 2812.9171) | 14107.5148 (21031.5182, 8274.9418) | 1234.3304 (1678.2662, 874.3808) |
| Bulgaria | 370.9360 (548.3372, 216.9548) | 397.5613 (577.6944, 228.9264) | 64.2677 (93.4068, 48.0675) | 14997.8195 (22724.8502, 8284.3609) | 18624.7198 (28354.7106, 10681.3683) | 1175.0884 (1706.3404, 872.3163) |
| Croatia | 131.1215 (190.5912, 74.7567) | 237.0146 (353.2954, 130.6474) | 28.4316 (36.4072, 20.9039) | 5986.8114 (9290.0348, 3287.9606) | 9404.5129 (14727.8375, 5294.9058) | 468.6940 (600.7801, 344.4137) |
| Czechia | 356.0564 (533.6052, 195.0575) | 631.0799 (937.9448, 352.2023) | 21.6231 (27.8328, 15.5998) | 14168.3083 (22031.1558, 7617.7341) | 24063.6388 (36950.7553, 13549.0944) | 370.8346 (475.8474, 272.5000) |
| Hungary | 346.6762 (502.6203, 199.2639) | 421.9270 (618.7620, 244.4998) | 29.2411 (43.7210, 20.8049) | 15660.5292 (23755.5772, 8717.4165) | 20306.7247 (31239.6056, 11387.7308) | 537.8748 (795.6739, 383.8663) |
| North Macedonia | 71.1758 (104.2247, 41.6974) | 181.8743 (270.1827, 102.5799) | 112.1339 (143.6987, 84.8333) | 2895.8666 (4292.6105, 1680.7204) | 7690.9481 (11543.2332, 4344.5891) | 1753.6767 (2265.4658, 1322.5447) |
| Montenegro | 13.8781 (20.2310, 8.1536) | 29.7688 (42.8679, 17.6393) | 82.2236 (139.5705, 56.0030) | 701.6529 (1065.1267, 403.3907) | 1458.5460 (2227.5322, 823.0208) | 1319.6058 (2225.0664, 906.2879) |
| Poland | 1083.9815 (1531.6125, 649.3867) | 1717.4623 (2472.3610, 1052.1217) | 29.9880 (38.7338, 22.8228) | 52378.7029 (78925.7099, 30503.2413) | 87001.1718 (133943.5464, 48959.5491) | 537.8214 (687.9272, 408.6593) |
| Romania | 364.5043 (528.2142, 212.2059) | 417.4558 (605.7117, 239.1500) | 38.0819 (54.3548, 27.3514) | 19617.9090 (29826.5305, 10880.2071) | 24866.0211 (38201.0118, 13580.0300) | 696.6962 (995.0285, 497.7036) |
| Serbia | 392.6207 (568.8938, 228.0919) | 718.6426 (1060.9594, 427.1501) | 73.0737 (99.8054, 53.8318) | 16683.3800 (25223.3156, 9948.6754) | 29988.6422 (45642.7755, 17271.2129) | 1228.8787 (1675.3007, 912.2915) |
| Slovakia | 130.4024 (188.0723, 73.0453) | 133.9132 (201.0050, 75.6920) | 36.5247 (46.9983, 27.0436) | 5636.3189 (8463.8160, 3032.2854) | 7965.8814 (12140.2065, 4471.5511) | 634.5610 (809.1734, 470.8683) |
| Slovenia | 49.7556 (71.1614, 28.2010) | 60.1647 (88.5878, 35.0637) | 9.5654 (12.7176, 6.7810) | 2280.3547 (3438.7524, 1227.0855) | 3113.1725 (4921.6155, 1724.6737) | 169.7816 (225.5418, 120.3206) |
| Belarus | 96.3059 (135.8172, 57.1299) | 99.2457 (148.4322, 54.0463) | 63.5393 (88.0308, 43.9902) | 5531.5059 (8438.5109, 3168.1324) | 7194.7037 (11721.2156, 3893.8158) | 1187.2083 (1642.7610, 815.2396) |
| Estonia | 11.9249 (18.6879, 5.4716) | 16.2314 (32.1197, 4.7832) | 5.3295 (10.5323, 1.6483) | 764.8745 (1319.4433, 353.4999) | 742.9852 (1460.0075, 212.7871) | 93.8126 (185.3615, 29.3270) |
| Latvia | 36.8403 (51.8980, 21.0198) | 70.5783 (107.6261, 37.9570) | 29.1494 (43.5034, 19.0818) | 1917.2346 (2942.9621, 1060.1457) | 2916.4740 (4565.3758, 1535.4234) | 526.4608 (794.7816, 348.0964) |
| Lithuania | 27.3821 (39.5090, 14.5984) | 57.4656 (89.2393, 29.6299) | 22.1411 (31.8059, 13.3251) | 1786.3238 (2822.1872, 942.7220) | 2669.9552 (4351.1403, 1320.7535) | 384.9489 (550.5026, 231.6669) |
| Republic of Moldova | 55.8381 (78.5264, 33.9439) | 84.2952 (127.1527, 46.5656) | 54.5418 (77.9266, 34.8976) | 3304.6324 (4946.5072, 1916.2926) | 5630.9073 (8882.8255, 2898.8071) | 1071.6904 (1523.2770, 686.2698) |
| Russian Federation | 1200.5010 (1744.2254, 651.8491) | 4387.0157 (7095.8665, 2151.8760) | 32.7946 (51.3169, 19.7922) | 79552.3810 (124907.4323, 41956.6685) | 168875.5721 (273763.3604, 79569.6292) | 627.7251 (983.9381, 374.1215) |
| Ukraine | 468.6360 (667.0925, 272.8516) | 313.7621 (514.2406, 150.8971) | 72.6376 (114.9186, 41.4813) | 32855.9041 (50449.3591, 17697.7400) | 39517.6202 (65497.2191, 19559.6459) | 1303.4824 (2010.9901, 749.5298) |
| Brunei Darussalam | 5.2435 (12.2562, 0.3802) | 9.9381 (19.8622, 1.9243) | 7.6838 (15.1519, 1.6084) | 166.5740 (384.6127, 11.5269) | 561.2773 (1124.1687, 112.1295) | 158.0348 (311.4226, 33.2569) |
| Japan | 1468.3455 (2577.4605, 321.0758) | 1324.0639 (2078.1173, 641.0472) | 5.5050 (8.2921, 2.9625) | 85180.7833 (158379.7990, 17126.2727) | 180913.3053 (313809.4870, 83123.1089) | 120.9197 (181.5853, 65.8124) |
| Republic of Korea | 1039.8189 (1509.8787, 535.9635) | 2080.3349 (2955.0045, 1210.2312) | 14.9626 (20.2345, 10.1144) | 46655.3118 (71700.6945, 23861.9561) | 162432.4957 (256592.5048, 90971.0155) | 278.8934 (371.6296, 192.2248) |
| Singapore | 68.1939 (99.0800, 38.0550) | 27.0460 (40.0710, 14.2642) | 9.5771 (14.2406, 5.2398) | 3717.9522 (5657.1378, 1951.3596) | 8981.7295 (15116.7283, 4404.6054) | 206.6314 (306.4227, 110.2368) |
| Australia | 139.9292 (351.7780, 5.1946) | 395.3798 (649.7540, 197.2153) | 4.5832 (6.7364, 2.6631) | 4467.9650 (11598.6065, 149.1424) | 16406.8404 (28060.2887, 7930.7935) | 82.1180 (119.2735, 48.2908) |
| New Zealand | 22.9968 (61.7998, 0.6591) | 40.8239 (75.0840, 14.5089) | 4.0579 (6.8274, 1.6161) | 936.8375 (2648.7077, 27.3814) | 2341.4546 (4473.1462, 780.5345) | 70.6259 (118.0624, 28.3482) |
| Andorra | 0.9945 (1.6538, 0.4292) | 1.2478 (2.1854, 0.5405) | 4.0096 (6.4437, 2.1114) | 33.3803 (55.1116, 15.0759) | 58.3443 (102.4537, 26.3089) | 72.3077 (116.0853, 38.1472) |
| Austria | 325.8977 (471.7685, 184.3014) | 237.1998 (355.0954, 135.9218) | 8.7376 (11.7990, 6.0883) | 7613.2986 (10971.1236, 4349.0784) | 7404.3913 (11741.5504, 3988.6027) | 153.1143 (206.8289, 107.5188) |
| Belgium | 331.0830 (469.8333, 189.1862) | 167.2641 (253.8252, 91.5517) | 5.7336 (7.5881, 3.9045) | 10210.2100 (15408.8172, 5794.4007) | 10693.9794 (17752.6955, 5495.4683) | 107.0845 (140.7211, 73.8497) |
| Cyprus | 76.9519 (116.9862, 38.9356) | 79.2104 (117.9672, 46.6296) | 17.8998 (23.7262, 12.8262) | 1775.9995 (2710.3196, 923.7731) | 2488.5904 (3716.0779, 1363.2829) | 277.3499 (368.6643, 198.3208) |
| Denmark | 135.0385 (204.2204, 66.8090) | 130.3318 (208.0566, 65.9434) | 4.9449 (7.0852, 2.9829) | 3720.4074 (5657.1820, 1826.6743) | 4119.2165 (6722.9994, 2016.3421) | 88.6640 (126.5091, 54.1366) |
| Finland | 39.9313 (79.9994, 7.0274) | 16.0303 (38.2356, 2.1745) | 2.1475 (4.6508, 0.2754) | 1874.6750 (3837.9400, 329.4051) | 1358.0870 (3245.0689, 183.9212) | 37.7767 (80.5367, 4.9843) |
| France | 1317.0486 (1992.2613, 670.7733) | 1267.6344 (1999.0773, 663.1468) | 3.7801 (5.2212, 2.4739) | 34298.0305 (52813.9305, 17307.2780) | 40025.0276 (65808.1981, 20407.1573) | 71.8912 (99.8603, 48.1929) |
| Germany | 3705.5518 (5371.5268, 2072.0176) | 2314.4481 (3503.7710, 1294.0931) | 7.7398 (10.6003, 5.2001) | 86437.4682 (126166.5764, 48458.7687) | 83645.2818 (135933.7414, 44546.0442) | 140.8123 (190.2581, 96.9758) |
| Greece | 217.4389 (308.3796, 124.5986) | 269.7959 (393.7920, 155.4016) | 16.1591 (20.2772, 12.2231) | 9680.4485 (14760.2029, 5549.5174) | 15053.7896 (23842.1089, 8246.2870) | 306.1395 (381.7916, 232.2126) |
| Iceland | 1.0586 (2.4324, 0.0722) | 0.7885 (1.9184, 0.1293) | 1.6739 (3.7304, 0.3082) | 41.5386 (97.4176, 3.1519) | 57.9102 (147.3729, 9.3565) | 29.3566 (64.3289, 5.4289) |
| Ireland | 61.8796 (101.1621, 24.7040) | 29.2354 (50.4740, 12.5412) | 3.8740 (6.0235, 1.9283) | 1947.2720 (3241.4726, 774.5287) | 1727.5422 (3114.7704, 709.7926) | 68.1265 (105.7396, 34.5012) |
| Israel | 177.1294 (250.8638, 101.6730) | 428.3657 (610.9388, 258.5154) | 9.6631 (12.1599, 7.2442) | 5510.2491 (7996.9995, 3190.0723) | 14022.1133 (21167.9337, 8085.8417) | 176.4695 (221.0980, 136.0573) |
| Italy | 3250.3112 (4797.0607, 1868.3968) | 2679.6537 (3915.8375, 1549.9441) | 9.1766 (11.7639, 6.6601) | 86619.2283 (126752.1078, 50211.0836) | 86274.2634 (135009.5372, 46974.1535) | 153.8687 (195.3999, 114.6355) |
| Luxembourg | 9.6914 (15.1313, 4.6397) | 5.6826 (9.4160, 2.6547) | 4.1417 (6.0810, 2.3032) | 304.4557 (491.1965, 146.9407) | 320.6162 (570.7462, 150.5214) | 71.7096 (104.6080, 40.4733) |
| Malta | 16.7145 (25.0657, 8.1458) | 19.6989 (30.0655, 10.9656) | 10.4604 (13.8755, 7.1941) | 420.4835 (639.1465, 211.1530) | 785.6986 (1232.1862, 431.0268) | 187.9363 (249.0474, 130.8298) |
| Netherlands | 671.0333 (975.6296, 374.1569) | 369.7546 (557.5184, 208.1354) | 6.1191 (8.1377, 4.2458) | 17414.9980 (25661.5963, 9850.2200) | 15190.3549 (24441.1540, 8005.9289) | 104.9250 (139.6553, 73.7513) |
| Norway | 56.6758 (99.3821, 17.9487) | 26.4361 (53.0929, 8.3340) | 1.9357 (3.3921, 0.7157) | 2370.9221 (4283.2336, 766.5741) | 1441.0319 (2911.3715, 435.6937) | 34.8116 (61.2813, 12.9714) |
| Portugal | 414.2741 (673.5355, 162.9242) | 283.0110 (477.5280, 129.2896) | 3.9396 (6.0127, 2.1534) | 12276.3425 (20341.3461, 4846.2178) | 11062.8707 (19894.2333, 4855.3799) | 72.3919 (109.9816, 39.8361) |
| Spain | 1512.8445 (2379.1580, 722.4343) | 842.3713 (1356.0738, 428.4590) | 4.0401 (5.6402, 2.5501) | 50476.7012 (81414.6886, 24333.4109) | 48936.6556 (83284.7675, 23161.7293) | 77.9411 (109.4130, 49.3907) |
| Sweden | 157.6580 (281.7687, 45.2898) | 78.0401 (159.7335, 19.6877) | 2.1526 (4.0554, 0.6318) | 5084.3234 (9345.3773, 1469.2653) | 3146.8416 (6791.0732, 768.0423) | 37.1312 (69.3878, 10.6427) |
| Switzerland | 267.2218 (405.0370, 140.0698) | 111.8774 (178.1250, 57.0606) | 4.0908 (5.7919, 2.5251) | 8174.5177 (12490.7051, 4267.5557) | 8038.5197 (13537.7978, 3832.8432) | 68.0471 (95.8992, 42.9593) |
| United Kingdom | 1400.7672 (2116.6290, 719.5713) | 598.4538 (933.2361, 314.7453) | 5.7483 (7.9712, 3.6325) | 47931.5402 (75708.1763, 24403.4523) | 56878.4470 (96440.4647, 28690.3994) | 108.8519 (152.6336, 69.7903) |
| Argentina | 1174.4106 (1846.4108, 605.8493) | 1281.5521 (1998.8349, 645.0051) | 12.5571 (19.9937, 6.4425) | 36206.8244 (57264.8845, 18337.9028) | 56696.1596 (93031.2305, 27924.9248) | 255.9207 (405.4527, 132.7236) |
| Chile | 286.7150 (415.1006, 167.2818) | 531.9521 (759.5604, 313.5101) | 15.2851 (20.7578, 10.1921) | 10650.5589 (15991.1243, 6116.7597) | 32930.5062 (52051.5089, 18863.6994) | 315.7187 (424.0916, 208.9159) |
| Uruguay | 89.5867 (159.4457, 34.9649) | 98.5368 (171.8729, 37.5653) | 8.9522 (15.4652, 3.4152) | 2631.9382 (4639.8137, 1092.4959) | 3852.4336 (7057.8207, 1404.7278) | 178.2190 (307.1658, 69.1835) |
| Canada | 425.3341 (760.0564, 113.1034) | 260.8357 (513.9462, 82.3729) | 2.2134 (3.9719, 0.8018) | 12046.6310 (21585.6905, 3192.6044) | 16789.2957 (34616.1879, 5137.3816) | 42.1269 (74.6921, 15.2787) |
| United States of America | 6455.5437 (10253.1520, 2594.2306) | 4213.8453 (7552.8633, 1738.5342) | 4.7405 (7.6714, 2.2893) | 234090.4802 (379160.0261, 92671.7702) | 284245.8441 (527822.6698, 111391.9701) | 95.0585 (152.5317, 45.7976) |
| Antigua and Barbuda | 5.8194 (9.6350, 1.5652) | 9.5844 (14.9270, 4.5962) | 23.1194 (41.0433, 8.3289) | 161.7135 (266.2176, 42.1460) | 387.0941 (603.8665, 176.6010) | 427.6894 (757.2212, 154.0519) |
| Bahamas | 11.0111 (17.9772, 2.5309) | 22.2932 (37.1408, 9.1932) | 21.1712 (40.4273, 7.6133) | 403.8759 (685.6351, 90.3954) | 1176.9393 (1954.7090, 453.7399) | 450.3609 (859.2197, 163.5935) |
| Barbados | 39.5304 (62.4440, 12.9005) | 57.3216 (87.2793, 29.4691) | 26.0136 (44.3556, 10.7140) | 1018.8979 (1621.1080, 333.9908) | 1879.2909 (2912.7693, 973.3390) | 478.5597 (818.5236, 198.4249) |
| Belize | 7.9888 (11.7707, 3.7365) | 28.4531 (41.8521, 16.0292) | 29.7301 (47.9176, 14.7463) | 251.1149 (377.8748, 113.1409) | 1197.0143 (1796.8337, 667.9450) | 606.0723 (974.4104, 302.4488) |
| Cuba | 353.1272 (546.1672, 140.8823) | 341.5367 (524.8974, 176.8009) | 29.3376 (47.8138, 14.5312) | 13498.7193 (21577.2286, 5474.0494) | 24644.2193 (40107.9442, 12082.4432) | 568.0419 (926.9811, 278.4956) |
| Dominica | 7.6216 (11.2210, 3.9660) | 10.2332 (16.1749, 5.0889) | 34.1816 (56.5007, 15.3625) | 216.4628 (330.9373, 112.3676) | 366.1329 (569.7839, 174.8389) | 638.7565 (1056.3406, 289.6760) |
| Dominican Republic | 133.2630 (201.2591, 70.1434) | 467.4061 (763.0128, 214.3602) | 38.9077 (67.7884, 14.7678) | 5984.3365 (9300.3889, 3226.4240) | 25849.8840 (40805.8409, 11758.4463) | 840.8996 (1460.8971, 316.4039) |
| Grenada | 11.6311 (17.5610, 5.1566) | 15.5184 (22.9856, 8.3054) | 37.4997 (65.9460, 15.0584) | 300.5434 (456.2666, 128.7774) | 584.7229 (901.2841, 301.9213) | 741.2823 (1310.8044, 292.1984) |
| Guyana | 48.3895 (75.3059, 20.7856) | 92.8969 (140.5899, 48.6595) | 63.8375 (104.0770, 30.0491) | 1875.3583 (2986.6853, 795.3337) | 4242.4461 (6436.0766, 2173.0858) | 1354.6128 (2209.8414, 644.6989) |
| Haiti | 487.6503 (729.4675, 283.8406) | 967.5429 (1561.0586, 550.3197) | 184.2158 (242.5222, 137.7048) | 17139.1203 (24976.0743, 9971.7747) | 42531.4614 (63714.9586, 24797.7352) | 3838.2212 (5116.2002, 2828.3755) |
| Jamaica | 211.8240 (318.1361, 118.3442) | 358.0170 (543.0945, 190.9610) | 24.0297 (36.3977, 13.2616) | 5729.4082 (8658.6135, 3223.6644) | 10691.4939 (16702.5170, 5734.2510) | 472.0594 (717.7990, 259.9274) |
| Saint Lucia | 14.1110 (20.9561, 6.8934) | 24.5915 (37.7857, 13.0607) | 25.7359 (42.9519, 11.0133) | 435.1725 (660.3542, 210.5701) | 1001.8799 (1559.4727, 509.2364) | 476.2150 (803.4334, 201.4356) |
| Saint Vincent and the Grenadines | 11.9358 (18.3255, 4.9839) | 17.3045 (26.6276, 8.7365) | 37.9786 (67.2608, 15.3084) | 344.3791 (528.2495, 145.0860) | 680.4257 (1043.9207, 340.9960) | 701.6912 (1239.0095, 281.3220) |
| Suriname | 17.9757 (26.5122, 9.8849) | 49.6084 (76.1054, 27.3689) | 45.0085 (73.8116, 20.9979) | 712.7261 (1086.6929, 380.5393) | 2592.5371 (3946.8829, 1428.4867) | 996.6290 (1611.8385, 466.7638) |
| Trinidad and Tobago | 172.4217 (275.0726, 46.3161) | 338.0804 (514.0225, 160.9979) | 36.8079 (67.0961, 13.2564) | 5432.3269 (8555.3045, 1466.1722) | 11954.6984 (18430.6405, 5795.1241) | 771.4591 (1403.1443, 276.5716) |
| Bolivia (Plurinational State of) | 234.7328 (344.5714, 136.2082) | 673.1867 (995.1266, 377.6911) | 39.3863 (60.6652, 26.1643) | 7577.9207 (10915.3738, 4477.8068) | 25187.9786 (38308.8076, 14167.0928) | 770.1474 (1193.8435, 509.2890) |
| Ecuador | 225.1837 (315.6383, 137.2543) | 760.4451 (1180.6100, 412.5800) | 20.0991 (29.9057, 12.2820) | 8519.6243 (12528.6716, 5169.4912) | 34761.0222 (53400.1598, 19512.7833) | 382.7377 (569.4795, 228.7185) |
| Peru | 322.3583 (460.6099, 196.5661) | 1131.7394 (1707.7750, 628.3705) | 19.9962 (27.7571, 13.8163) | 12050.5661 (17648.9346, 7211.9675) | 45612.3212 (67460.7322, 26057.3562) | 424.2330 (589.6563, 295.8513) |
| Colombia | 637.8152 (906.0619, 384.2949) | 1063.9858 (1626.4637, 564.4990) | 15.5616 (22.5196, 9.9723) | 30004.1134 (45442.4592, 17483.3416) | 67520.6122 (106205.9249, 37188.6211) | 304.2254 (439.6513, 195.1085) |
| Costa Rica | 45.4630 (65.6308, 25.9593) | 146.8911 (217.9205, 84.2012) | 10.1764 (14.4426, 6.6586) | 2399.0148 (3606.6776, 1320.1484) | 8294.1285 (13359.9956, 4488.8129) | 211.8793 (297.8162, 138.4656) |
| El Salvador | 125.9383 (177.8105, 75.3460) | 467.9641 (699.6132, 247.6011) | 26.6249 (37.9657, 16.2886) | 4939.9903 (7173.4117, 2899.8566) | 17153.0626 (26109.9527, 9472.4726) | 546.0041 (785.2042, 336.9307) |
| Guatemala | 111.0044 (153.6996, 68.8112) | 1286.1113 (1866.3458, 762.6421) | 46.0636 (59.8701, 30.5106) | 5502.7030 (8099.5754, 3252.9629) | 51917.6836 (75352.9243, 30782.7652) | 844.0794 (1105.6659, 569.0582) |
| Honduras | 58.3004 (84.0941, 35.4935) | 322.1817 (504.5109, 184.4556) | 96.7109 (124.3105, 72.6763) | 3334.8000 (4887.5869, 1952.0507) | 17857.8729 (27147.3062, 9943.8942) | 1825.6377 (2315.5999, 1376.2639) |
| Mexico | 5429.6290 (7666.8096, 3404.0336) | 12700.8318 (19440.2580, 7416.3252) | 21.9106 (31.0777, 13.9528) | 198280.1354 (283208.5700, 119412.8310) | 467038.5003 (709454.5226, 259003.0976) | 415.9076 (594.3744, 265.7043) |
| Nicaragua | 68.9200 (98.6751, 40.7956) | 242.4784 (371.3152, 141.1249) | 35.8285 (46.1877, 26.3842) | 3195.6699 (4618.8219, 1861.1608) | 12965.8718 (19824.3053, 7399.3168) | 692.0105 (893.0032, 507.8429) |
| Panama | 52.2482 (77.2919, 28.9953) | 156.7315 (255.5083, 76.3608) | 9.8402 (15.9647, 5.3026) | 2158.2590 (3320.8040, 1180.1044) | 6904.2495 (11316.2163, 3446.5430) | 197.5722 (320.5079, 104.7736) |
| Venezuela (Bolivarian Republic of) | 552.2190 (805.6729, 295.3944) | 1848.6650 (3029.5899, 990.0723) | 26.7084 (41.5188, 14.8101) | 20448.6511 (30371.7048, 10383.8983) | 69470.7604 (110353.8876, 37293.5850) | 554.1965 (871.6530, 308.5544) |
| Brazil | 4047.4072 (6276.3264, 1863.1585) | 7902.6957 (12709.9925, 3908.8747) | 13.6584 (19.6412, 7.9680) | 155075.0852 (245799.0385, 74811.4709) | 324493.4088 (543390.9820, 165731.2963) | 305.5272 (438.7638, 178.9598) |
| Paraguay | 92.5886 (136.5859, 53.3950) | 401.9497 (702.3332, 164.8962) | 24.2970 (44.0843, 7.9649) | 3365.9494 (5025.3669, 1911.5081) | 13678.7929 (23737.8631, 5599.1216) | 494.7849 (899.8803, 164.0100) |
| Algeria | 201.7886 (309.3525, 114.1027) | 1008.2023 (1532.5450, 579.0356) | 68.7873 (93.2150, 46.2354) | 12013.0696 (18533.8461, 6947.6569) | 78445.2654 (120961.6215, 44020.6249) | 1119.9920 (1525.5738, 743.4732) |
| Bahrain | 25.5228 (36.2368, 15.3532) | 134.5762 (193.5043, 78.8083) | 95.1705 (116.4740, 74.6441) | 885.0581 (1269.7308, 525.4651) | 6281.8848 (9373.7525, 3732.3873) | 1618.2532 (1987.1595, 1269.3713) |
| Egypt | 1303.9920 (1802.6440, 789.8392) | 5510.7530 (8204.9409, 3340.9740) | 208.9764 (259.4060, 160.8278) | 44787.7949 (63465.8956, 27349.0486) | 233546.2041 (344771.6940, 140729.7331) | 4028.0615 (5048.7465, 3098.6816) |
| Iran (Islamic Republic of) | 539.1453 (775.6628, 316.4576) | 2872.9221 (3996.9189, 1759.5291) | 58.2497 (70.1062, 45.6383) | 25981.0985 (38514.3084, 14936.1269) | 156407.9341 (235484.0394, 90133.3214) | 1085.1715 (1295.1876, 871.0817) |
| Iraq | 592.2606 (870.8443, 340.5141) | 1898.3243 (2764.0101, 1113.4815) | 126.6120 (166.2002, 90.8291) | 23406.3545 (33965.5468, 13719.0822) | 111457.2354 (166979.7637, 64740.1745) | 2428.5555 (3215.1441, 1705.3618) |
| Jordan | 112.1446 (165.0203, 70.8419) | 440.1257 (640.1835, 255.3377) | 43.8885 (57.3135, 32.1822) | 4200.9303 (6152.0297, 2509.9299) | 27694.4868 (42371.5130, 15796.7801) | 856.5456 (1110.9491, 629.9808) |
| Kuwait | 20.2319 (28.3373, 12.6222) | 112.3621 (164.7849, 66.4322) | 53.3763 (69.3310, 39.6490) | 1416.6448 (2181.8765, 818.3139) | 11946.2709 (19008.9518, 6745.2385) | 1116.1646 (1440.1118, 829.9152) |
| Lebanon | 117.0708 (182.1354, 64.8324) | 346.1505 (527.5094, 191.9493) | 25.4873 (36.5681, 16.4446) | 4384.6205 (6870.8002, 2511.8222) | 16524.3417 (25833.4069, 9128.0362) | 484.7730 (691.4292, 308.1880) |
| Libya | 44.9455 (66.8325, 25.6448) | 225.4167 (350.2492, 118.2306) | 74.3996 (102.3484, 52.1648) | 2286.0430 (3435.7441, 1328.9104) | 15613.8281 (24190.7975, 9033.3934) | 1549.7296 (2141.8640, 1081.3299) |
| Morocco | 281.8790 (431.2241, 167.1045) | 1361.0755 (2029.1171, 746.5633) | 77.3702 (100.7701, 54.1477) | 16901.4797 (26279.1034, 9631.3199) | 101543.2160 (158715.2134, 57361.8142) | 1498.2624 (1987.2542, 1053.2220) |
| Palestine | 77.4309 (115.5963, 43.0203) | 208.9249 (307.9970, 125.5800) | 77.1779 (100.2340, 53.1284) | 2226.4341 (3335.1405, 1297.8937) | 8972.5632 (13172.0154, 5262.9705) | 1375.0546 (1784.7667, 943.2021) |
| Oman | 45.7038 (71.5696, 26.1140) | 138.4882 (207.2651, 81.9611) | 85.2060 (109.5421, 60.8069) | 1657.5870 (2532.1948, 954.8259) | 7265.2232 (10895.1077, 4155.6960) | 1616.7366 (2067.7694, 1135.3169) |
| Qatar | 11.0893 (16.0925, 6.4201) | 75.8500 (112.6771, 42.5905) | 76.5255 (98.6019, 55.8202) | 467.3531 (682.2914, 269.3297) | 6040.6504 (9447.0626, 3435.7718) | 1334.9401 (1748.3379, 968.0807) |
| Saudi Arabia | 239.1683 (375.5158, 129.9935) | 999.4598 (1500.0335, 573.5267) | 108.3596 (135.1995, 82.6149) | 11031.7820 (16694.3984, 6434.1168) | 74012.8677 (112637.0183, 42370.8111) | 2357.9948 (2965.3217, 1752.3638) |
| Syrian Arab Republic | 158.4789 (233.4414, 94.4907) | 425.0902 (660.4821, 232.4855) | 109.8995 (149.4290, 76.1267) | 7167.5804 (10651.0251, 4191.6334) | 28110.7474 (42839.3347, 16095.0668) | 2071.7420 (2866.9920, 1404.1424) |
| Tunisia | 83.9945 (125.1219, 50.4974) | 393.7593 (613.9018, 211.6866) | 52.0998 (74.2826, 34.3125) | 4718.9541 (7124.5574, 2670.7948) | 28294.1330 (44832.0525, 15425.0388) | 959.3440 (1358.5252, 635.2005) |
| Turkey | 2033.8575 (3017.1106, 1191.3605) | 4398.5121 (6479.2045, 2612.2398) | 43.6742 (54.8425, 32.6723) | 61023.6167 (88990.1697, 35392.0898) | 189983.5987 (284261.4939, 110571.5898) | 790.0876 (992.7786, 583.3829) |
| United Arab Emirates | 27.4166 (41.3472, 15.6881) | 163.4898 (244.9808, 91.9335) | 83.1838 (106.1851, 61.2863) | 1220.0506 (1790.8846, 700.6412) | 15742.0067 (24355.6407, 8882.8718) | 1463.4579 (1858.5294, 1070.5401) |
| Yemen | 101.4778 (161.8507, 56.6916) | 353.3728 (579.2117, 195.5289) | 153.4705 (208.0587, 107.6818) | 4861.8468 (7531.2030, 2853.5175) | 23692.1541 (36030.3556, 13252.2573) | 3113.7598 (4279.1035, 2193.1777) |
| Afghanistan | 326.6799 (504.7764, 186.7852) | 674.1217 (1029.5622, 366.1005) | 208.9015 (261.4788, 162.9161) | 14289.9565 (21464.3812, 8367.1481) | 46512.9688 (70820.0091, 27467.5516) | 4510.8856 (5737.1083, 3510.7571) |
| Bangladesh | 2212.4934 (3239.7269, 1342.4960) | 7829.1550 (11795.3774, 4498.1711) | 117.7515 (145.9221, 90.6931) | 72670.0798 (106761.1355, 42561.3994) | 304027.0050 (460527.9072, 179699.1283) | 2400.4441 (3023.0086, 1821.9295) |
| Bhutan | 9.0819 (14.1356, 5.0510) | 34.3140 (51.5689, 19.5160) | 52.6397 (68.0802, 37.5600) | 357.9122 (535.4331, 210.4059) | 1223.9470 (1857.3724, 694.4295) | 1080.8536 (1420.7495, 763.5137) |
| India | 17257.5697 (24354.8863, 10460.3304) | 64451.9819 (92074.8951, 38805.8162) | 89.2817 (105.5707, 73.6045) | 691496.1496 (993452.1642, 416941.0501) | 2585950.0419 (3790292.2545, 1519892.9845) | 2006.3881 (2385.2870, 1664.0329) |
| Nepal | 325.9742 (480.6613, 191.4634) | 1279.4469 (1898.2655, 729.2639) | 100.4017 (126.2418, 76.6951) | 13992.3546 (20704.2116, 8252.9111) | 56892.2890 (85391.8361, 32593.3592) | 2092.8937 (2656.1850, 1582.4584) |
| Pakistan | 2717.8095 (4062.3049, 1546.2243) | 9066.3214 (13484.9369, 5530.6916) | 118.3504 (148.1422, 92.4633) | 96109.2863 (141660.1942, 56052.8876) | 398709.5773 (594041.1373, 226508.4594) | 2593.1266 (3243.5345, 2040.9441) |
| Angola | 317.5947 (481.2215, 184.0123) | 908.6813 (1382.5562, 506.7550) | 81.1427 (109.5948, 55.5353) | 11541.4811 (17273.3069, 6716.7859) | 40640.5044 (62275.0207, 23014.2730) | 1658.0742 (2225.1289, 1112.9306) |
| Central African Republic | 122.9380 (184.9129, 75.4565) | 233.6140 (370.5148, 131.9017) | 166.3625 (219.2525, 123.3461) | 4365.2221 (6540.8436, 2663.2537) | 10464.2143 (15879.2106, 6246.4660) | 3603.5872 (4847.3428, 2627.9793) |
| Congo | 121.7449 (180.7077, 73.6836) | 282.8568 (419.5145, 167.3049) | 113.5175 (142.3845, 84.7032) | 3884.3352 (5660.8870, 2317.5648) | 11090.6798 (16453.3209, 6720.4355) | 2323.9607 (2998.2185, 1713.9529) |
| Democratic Republic of the Congo | 1283.3086 (1872.2272, 775.3556) | 3031.1428 (4534.2326, 1746.7475) | 118.8622 (154.6945, 89.2108) | 42181.0348 (61068.8146, 25599.5327) | 119047.6569 (177064.1351, 68549.5852) | 2437.6038 (3181.4837, 1820.5656) |
| Equatorial Guinea | 19.0049 (28.4986, 11.0196) | 53.9209 (89.3466, 29.7073) | 64.9340 (104.4058, 35.3572) | 625.5251 (939.1657, 358.5511) | 2170.7930 (3492.7627, 1249.0844) | 1281.1649 (2081.1214, 686.6784) |
| Gabon | 65.0120 (100.8986, 35.2212) | 129.5244 (203.9342, 73.6374) | 57.7367 (85.7214, 34.0817) | 1908.1494 (2933.9383, 1070.2289) | 4709.4158 (7244.5961, 2737.1956) | 1150.5566 (1739.9938, 690.6629) |
| Burundi | 218.4608 (329.3708, 122.6929) | 353.2324 (571.3170, 191.8183) | 109.9592 (138.6430, 86.0930) | 6363.8609 (9595.0818, 3592.1073) | 12239.2796 (19044.1649, 6779.5229) | 2356.5567 (2925.3453, 1840.9389) |
| Comoros | 14.4149 (21.6734, 8.1873) | 33.4862 (50.8277, 16.3266) | 80.4811 (104.6552, 61.2708) | 460.3594 (681.8692, 263.8857) | 1195.0878 (1786.1894, 631.5106) | 1686.1038 (2174.5232, 1270.0797) |
| Djibouti | 7.6332 (11.6698, 4.3578) | 45.5436 (73.0444, 25.0374) | 71.4120 (101.5595, 45.9993) | 255.3485 (389.6242, 146.5434) | 1648.2975 (2572.5625, 969.7007) | 1512.4582 (2166.4702, 978.3977) |
| Eritrea | 92.7411 (143.6804, 55.0805) | 238.2680 (363.8865, 131.8553) | 108.5166 (138.9726, 81.3550) | 3320.6937 (5105.1454, 1941.8744) | 9148.4173 (13927.4880, 5319.3078) | 2347.6193 (3042.1907, 1741.3939) |
| Ethiopia | 2263.3344 (3277.9682, 1366.4842) | 2749.2353 (4006.0334, 1664.2427) | 64.0707 (78.1060, 50.1699) | 73252.8113 (106275.8691, 43638.8221) | 96813.4999 (138839.2534, 57437.9367) | 1358.7548 (1653.2710, 1079.2226) |
| Kenya | 371.9059 (548.4099, 218.7473) | 1355.2978 (1990.1332, 799.6427) | 72.5461 (94.2301, 55.1268) | 10920.1482 (15906.7866, 6347.2654) | 43301.1534 (63672.3809, 25885.4830) | 1452.5802 (1849.7596, 1127.6796) |
| Madagascar | 304.0566 (457.6339, 182.1824) | 621.9556 (918.9441, 346.3697) | 147.6125 (190.8167, 110.9566) | 9273.2390 (13739.2970, 5495.1666) | 23882.4343 (34875.9835, 13589.5904) | 3268.4796 (4224.5645, 2468.2997) |
| Malawi | 284.4605 (418.5451, 174.1693) | 601.4619 (876.4106, 354.6389) | 116.1210 (142.6192, 93.0579) | 8317.3039 (12180.6741, 5109.3162) | 18806.9443 (27680.5189, 11013.3908) | 2494.8429 (3037.8389, 1991.7974) |
| Mauritius | 33.1161 (60.1216, 12.4378) | 172.1431 (310.3145, 51.9253) | 11.1003 (19.6069, 3.7956) | 1235.8737 (2268.5540, 461.6804) | 5719.3806 (10346.5130, 1848.4585) | 244.5813 (435.8394, 85.1886) |
| Mozambique | 392.4394 (579.3518, 235.3091) | 955.7304 (1470.8650, 536.5722) | 132.7362 (167.6160, 99.6203) | 11975.9439 (17694.9956, 7077.9024) | 34421.2278 (52159.9196, 19264.0044) | 2981.5765 (3769.3920, 2212.2942) |
| Rwanda | 281.9018 (431.4773, 157.8600) | 430.3934 (697.2839, 207.7849) | 95.3285 (125.5875, 69.9814) | 8495.0032 (12931.5742, 4780.0803) | 13667.8524 (21489.0332, 7022.7382) | 1938.8210 (2523.7979, 1436.5212) |
| Seychelles | 0.8005 (1.5214, 0.2281) | 2.4150 (4.4788, 0.8757) | 11.1875 (20.7051, 3.9405) | 33.2461 (65.4734, 9.0879) | 165.6978 (310.2037, 54.7717) | 235.9221 (430.5276, 84.3212) |
| Somalia | 207.3518 (307.0246, 118.0206) | 539.5848 (821.1319, 299.5288) | 107.9733 (146.6048, 77.5545) | 6954.4856 (10309.6469, 3957.4051) | 19999.3759 (29878.2788, 11335.9243) | 2428.3496 (3349.6723, 1732.4733) |
| United Republic of Tanzania | 683.1663 (981.6384, 417.5554) | 1656.9912 (2473.7456, 931.0888) | 97.7375 (124.7475, 75.3173) | 19690.4859 (28278.0740, 11809.5806) | 54203.7227 (80046.6194, 30360.6371) | 2026.5536 (2600.3746, 1553.2857) |
| Uganda | 453.3310 (801.1458, 233.2968) | 1139.0282 (1852.9529, 597.3356) | 83.4900 (106.1974, 63.7702) | 12961.8043 (21860.6580, 7032.5818) | 37230.6098 (58649.1087, 20368.7863) | 1772.2990 (2262.4977, 1350.7746) |
| Zambia | 232.2742 (345.2900, 141.7503) | 541.1695 (819.3035, 310.4794) | 117.0294 (149.1036, 85.3011) | 7461.2021 (11027.4459, 4497.5206) | 21900.7298 (32795.8681, 12845.8153) | 2438.0755 (3128.9163, 1753.5048) |
| Botswana | 53.6251 (81.5719, 30.3939) | 132.9284 (196.2813, 75.1739) | 36.0919 (61.0156, 20.6290) | 1546.5058 (2336.3271, 893.8686) | 4417.9733 (6484.8002, 2483.0687) | 728.3198 (1217.3262, 414.1072) |
| Lesotho | 70.1028 (105.9644, 40.0310) | 184.4991 (277.2367, 102.3278) | 116.8003 (154.8225, 83.3770) | 1864.7876 (2723.9048, 1070.4511) | 5530.5240 (8275.3888, 3127.6298) | 2480.0005 (3423.4379, 1715.3724) |
| Namibia | 65.5169 (97.0152, 37.5678) | 160.3283 (248.0899, 89.0026) | 66.3481 (104.7590, 36.3912) | 1864.2316 (2815.6288, 1062.9833) | 4821.2758 (7370.6045, 2762.9597) | 1318.0783 (2120.7114, 721.6490) |
| South Africa | 1514.9466 (2161.3659, 919.5104) | 5669.1546 (8300.3453, 3446.2703) | 37.6212 (48.7134, 26.9322) | 48130.6642 (69122.3870, 29294.9553) | 177801.7359 (258013.5438, 108007.1256) | 762.0346 (991.5964, 549.3509) |
| Eswatini | 39.3410 (58.5744, 22.8852) | 114.0255 (177.3668, 64.8844) | 81.1937 (122.5299, 45.1891) | 1125.9988 (1675.0198, 652.8646) | 3615.7915 (5603.4015, 2077.4461) | 1728.9164 (2718.2571, 937.5876) |
| Zimbabwe | 254.0565 (377.0508, 151.7926) | 755.5897 (1116.6165, 447.6072) | 115.9944 (144.9624, 90.1198) | 7552.7843 (11143.8420, 4615.9282) | 25173.7132 (37115.6111, 15254.3699) | 2433.7836 (3084.4139, 1866.1092) |
| Benin | 99.5009 (145.7795, 60.4466) | 318.5175 (480.5260, 184.5778) | 109.4929 (133.1350, 88.4789) | 3395.6314 (5050.4336, 1992.9204) | 14714.7625 (22341.5221, 8416.9717) | 2173.4567 (2672.1201, 1715.7865) |
| Burkina Faso | 251.9308 (373.1966, 148.4326) | 528.9346 (806.0034, 309.2748) | 97.9756 (119.7836, 77.7982) | 7870.5268 (11593.4088, 4573.2521) | 21409.4398 (32642.5387, 12604.1611) | 1986.6468 (2466.6153, 1558.5470) |
| Cameroon | 285.6226 (419.9902, 164.6622) | 1063.5099 (1654.6043, 601.7117) | 112.0362 (150.1683, 83.8881) | 9136.0320 (13477.3587, 5297.9722) | 40523.8018 (61712.0159, 23831.4812) | 2308.9673 (3133.6259, 1711.6712) |
| Cabo Verde | 5.1389 (7.3606, 3.0866) | 29.9050 (43.2972, 17.0968) | 76.8837 (100.7830, 55.8253) | 214.8514 (316.2146, 124.0399) | 1182.7449 (1729.5180, 685.4017) | 1466.3766 (1927.9065, 1065.5645) |
| Chad | 115.6946 (175.8857, 66.9406) | 348.2188 (544.6337, 194.8674) | 128.4862 (163.6680, 98.3066) | 3889.0315 (5863.3396, 2257.7203) | 14583.4517 (22024.3948, 8645.2106) | 2712.3037 (3500.9816, 2064.6399) |
| C么te d'Ivoire | 201.4482 (295.7162, 118.4188) | 753.6355 (1151.4424, 434.0900) | 118.4967 (153.5740, 91.0513) | 7657.8759 (11427.5457, 4486.6713) | 32647.6065 (48791.7190, 19249.2828) | 2446.2834 (3196.4066, 1867.8162) |
| Gambia | 15.7651 (23.5820, 9.0652) | 73.7386 (114.5369, 40.7396) | 149.6208 (184.8633, 114.9117) | 562.3702 (855.5627, 330.3634) | 2797.0133 (4251.1864, 1622.1978) | 3055.6715 (3806.9986, 2330.8860) |
| Ghana | 278.8483 (415.2742, 158.6914) | 1340.1942 (2002.2864, 781.1918) | 126.9257 (158.1699, 96.4251) | 10318.5082 (15207.0880, 6119.3565) | 52162.5447 (77513.4740, 30948.5959) | 2651.1003 (3322.5865, 1973.3984) |
| Guinea | 170.1514 (265.7469, 97.9996) | 400.3419 (624.9934, 227.5020) | 129.8313 (165.4750, 100.6783) | 5281.3434 (7771.7834, 3019.1439) | 14424.0037 (22105.0812, 8458.0293) | 2700.3277 (3477.0362, 2049.9523) |
| Guinea-Bissau | 28.9004 (44.2420, 17.0392) | 63.3658 (94.7035, 34.8580) | 183.8848 (226.4897, 142.0107) | 1006.0662 (1514.4583, 597.0846) | 2690.0891 (3961.6450, 1564.8170) | 3943.7459 (4931.7391, 3026.1279) |
| Liberia | 61.6026 (89.9970, 36.2852) | 152.4990 (233.1185, 83.9952) | 126.1362 (164.6433, 97.2257) | 2021.1092 (2999.4014, 1221.8369) | 6633.7532 (9985.3149, 3878.1205) | 2581.8157 (3357.9213, 1960.2450) |
| Mali | 214.4570 (318.2347, 128.2993) | 603.6235 (909.4482, 345.0797) | 91.0917 (112.6697, 71.5652) | 8691.3109 (12840.5078, 5202.3021) | 30578.2518 (46303.7577, 17644.2419) | 1900.0485 (2367.7718, 1483.1656) |
| Mauritania | 52.5933 (78.1882, 30.6761) | 137.8914 (211.2805, 76.9418) | 108.5723 (140.8426, 81.6530) | 1611.4063 (2387.5316, 948.0302) | 4487.7512 (6657.8943, 2632.1851) | 2137.6030 (2800.0276, 1583.7662) |
| Niger | 97.1713 (148.9181, 56.5030) | 360.3050 (567.1940, 200.5148) | 103.5324 (132.8116, 79.1080) | 3890.5318 (5873.2358, 2330.5408) | 16950.2716 (25131.3929, 9924.0432) | 2098.1312 (2701.5127, 1577.5347) |
| Nigeria | 2658.2764 (3835.6433, 1574.2570) | 5511.3862 (8077.2026, 3200.8968) | 89.4765 (110.6697, 72.0616) | 80541.0343 (116573.1608, 46930.4409) | 197035.2526 (289995.2671, 118286.5238) | 1736.8735 (2201.8880, 1364.7259) |
| Sao Tome and Principe | 1.5111 (2.2088, 0.8951) | 3.2930 (4.8695, 1.8921) | 79.9281 (98.4550, 62.8038) | 64.3425 (96.2794, 37.6636) | 203.6007 (310.5485, 114.0248) | 1630.8876 (2008.7385, 1271.8439) |
| Senegal | 173.2960 (255.4964, 103.8636) | 607.1229 (919.2789, 347.7347) | 125.3544 (160.9772, 97.4527) | 6367.2086 (9579.4199, 3715.8342) | 24910.9196 (37186.3703, 14900.5905) | 2509.9277 (3216.4996, 1930.5661) |
| Sierra Leone | 87.7071 (129.7857, 52.3773) | 211.7352 (327.1017, 120.8480) | 137.0845 (171.6746, 106.6510) | 2927.1292 (4383.0847, 1746.5075) | 9054.0220 (13799.0499, 5104.3731) | 2852.9951 (3646.1736, 2145.3955) |
| Togo | 50.0746 (74.7731, 29.0407) | 218.0073 (343.4568, 126.3062) | 134.4909 (172.4491, 102.4317) | 1733.8464 (2584.1240, 1036.6798) | 8477.2909 (12899.1867, 4935.6715) | 2788.4758 (3638.1158, 2093.6717) |
| American Samoa | 1.0263 (3.1461, 0.0000) | 3.1157 (6.9820, 0.3543) | 15.3770 (34.9506, 1.6332) | 39.1262 (116.9186, 0.0000) | 133.0027 (288.0187, 15.7445) | 345.6997 (798.7835, 36.6436) |
| Bermuda | 1.7373 (4.2421, 0.0000) | 1.4633 (2.8115, 0.3066) | 4.1944 (8.0229, 0.7757) | 52.8445 (128.6255, 0.0000) | 65.6673 (128.3286, 14.0289) | 79.1729 (150.6587, 14.7156) |
| Cook Islands | 1.1514 (2.7973, 0.0484) | 1.4190 (3.6437, 0.0000) | 5.9663 (14.4508, 0.0000) | 36.1444 (88.7314, 1.4905) | 48.5053 (123.0434, 0.0000) | 132.8978 (325.4725, 0.0000) |
| Greenland | 0.2681 (0.6765, 0.0014) | 0.2662 (0.7094, 0.0100) | 4.8798 (12.8951, 0.1886) | 8.4481 (21.3176, 0.0423) | 15.4520 (41.8451, 0.5360) | 98.3137 (262.2077, 3.7354) |
| Guam | 2.1624 (5.4379, 0.0000) | 4.0658 (6.7192, 1.8885) | 12.9406 (20.0802, 6.2077) | 84.3264 (212.6969, 0.0000) | 243.0341 (417.0123, 106.6843) | 359.3365 (562.9593, 172.4891) |
| Monaco | 0.2989 (0.6053, 0.0495) | 0.4127 (0.6762, 0.1884) | 6.3894 (10.2486, 3.1465) | 11.8337 (23.0775, 1.9884) | 25.8612 (44.1465, 11.5318) | 115.1081 (183.6456, 57.7978) |
| Nauru | 0.2280 (0.7716, 0.0000) | 0.3643 (0.9075, 0.0422) | 27.3783 (60.1100, 3.5851) | 8.4187 (28.4951, 0.0000) | 14.7100 (35.7028, 1.7298) | 671.5340 (1504.5798, 85.9435) |
| Niue | 0.1910 (0.3678, 0.0845) | 0.1452 (0.3350, 0.0173) | 17.7182 (38.8345, 1.7160) | 5.4535 (10.5018, 2.4408) | 4.9468 (11.3928, 0.6356) | 382.6371 (850.9881, 36.0419) |
| Northern Mariana Islands | 0.8590 (2.0980, 0.0000) | 2.8986 (4.7745, 1.2766) | 19.2017 (30.7486, 9.1469) | 36.1423 (88.5960, 0.0000) | 121.9614 (206.9263, 53.6636) | 420.5273 (668.7584, 200.1078) |
| Palau | 0.4981 (1.2924, 0.0013) | 1.4120 (2.9798, 0.1287) | 17.9166 (37.5316, 1.9394) | 17.6585 (45.7614, 0.0481) | 59.6899 (126.6174, 5.2648) | 405.2968 (853.5952, 44.6570) |
| Puerto Rico | 96.9417 (304.2205, 0.0022) | 157.6422 (310.3452, 36.8237) | 2.5804 (4.8326, 0.6632) | 3099.9973 (9528.5302, 0.0723) | 5902.1588 (11727.8406, 1366.9289) | 55.2893 (101.9902, 14.0423) |
| Saint Kitts and Nevis | 2.6621 (5.4068, 0.5570) | 2.1631 (3.9566, 0.6899) | 10.0155 (17.3700, 3.6034) | 75.7091 (154.0780, 15.5262) | 100.5032 (189.6157, 34.0766) | 186.9923 (328.6249, 67.2579) |
| San Marino | 0.4434 (0.7736, 0.1667) | 0.4019 (0.7245, 0.1803) | 3.5037 (5.8211, 1.7397) | 13.7942 (23.7860, 5.1309) | 24.8907 (44.7337, 11.0440) | 66.5349 (108.3202, 33.2279) |
| Tokelau | 0.0524 (0.1695, 0.0005) | 0.0588 (0.1533, 0.0001) | 10.7738 (26.8658, 0.0909) | 1.7329 (5.5546, 0.0151) | 2.2530 (5.9328, 0.0047) | 237.0068 (599.8933, 2.0018) |
| Tuvalu | 0.6855 (1.1583, 0.3627) | 0.5334 (1.2559, 0.1339) | 40.1543 (62.6920, 23.5145) | 22.4715 (37.7152, 11.8333) | 18.9298 (43.7844, 4.8318) | 922.8931 (1445.7003, 535.0659) |
| United States Virgin Islands | 2.2470 (5.8943, 0.0565) | 3.5809 (6.2167, 1.3462) | 7.2552 (11.9763, 3.2343) | 95.8892 (254.9247, 2.4548) | 214.4550 (388.1002, 81.4758) | 139.3734 (229.2894, 61.4318) |
| South Sudan | 210.5267 (324.2065, 114.4608) | 368.9654 (591.7352, 200.3655) | 101.7101 (135.9572, 75.6316) | 5905.1786 (8950.6868, 3268.0046) | 12124.0573 (19259.3667, 6834.2832) | 2217.1076 (3009.7320, 1604.1855) |
| Sudan | 204.6175 (317.7072, 123.1224) | 626.4669 (945.8173, 361.2674) | 152.4323 (201.5528, 114.1875) | 9767.4430 (14701.8529, 5801.4550) | 41054.8566 (62691.4391, 23860.5248) | 3137.8653 (4240.7251, 2307.9984) |
